# Bottom-up Assembled Synthetic SARS-CoV-2 Miniviruses Reveal Lipid Membrane Affinity of Omicron Variant Spike Glycoprotein

**DOI:** 10.1021/acsnano.3c08323

**Published:** 2023-11-17

**Authors:** Ana Yagüe Relimpio, Andreas Fink, Duc Thien Bui, Sebastian Fabritz, Martin Schröter, Alessia Ruggieri, Ilia Platzman, Joachim P. Spatz

**Affiliations:** † Department for Cellular Biophysics, Max Planck Institute for Medical Research, Jahnstrasse 29, 69120 Heidelberg, Germany; ‡ Institute for Molecular Systems Engineering and Advanced Materials (IMSEAM), Heidelberg University, Im Neuenheimer Feld 225, 69120 Heidelberg, Germany; § Department for Chemical Biology, Max Planck Institute for Medical Research, Jahnstrasse 29, 69120 Heidelberg, Germany; ∥ Heidelberg University, Medical Faculty, Centre for Integrative Infectious Disease Research (CIID), Department of Infectious Diseases, Molecular Virology, Im Neuenheimer Feld 344, 69120 Heidelberg, Germany; ⊥ Max Planck-Bristol Center for Minimal Biology, University of Bristol, 1 Tankard’s Close, Bristol BS8 1TD, U.K.; # Max Planck School Matter to Life, Jahnstrasse 29, 69120 Heidelberg, Germany

**Keywords:** giant unilamellar vesicles, bottom-up synthetic biology, SARS-CoV-2, Omicron, small unilamellar vesicles, spike protein, synthetic minivirus

## Abstract

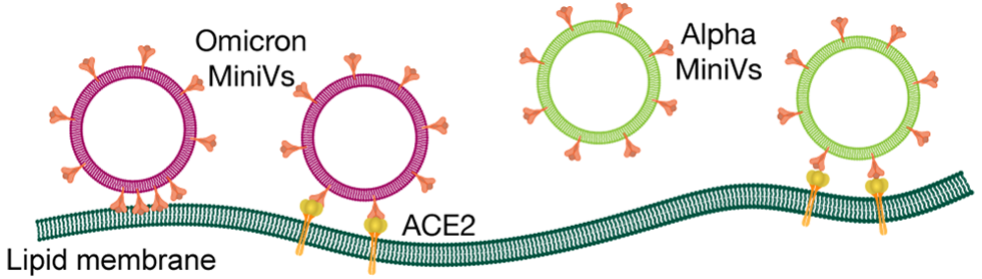

The ongoing COVID-19 pandemic has been brought on by severe acute respiratory syndrome coronavirus 2 (SARS-CoV-2). The spike glycoprotein (S), which decorates the viral envelope forming a corona, is responsible for the binding to the angiotensin-converting enzyme 2 (ACE2) receptor and initiating the infection. In comparison to previous variants, Omicron S presents additional binding sites as well as a more positive surface charge. These changes hint at additional molecular targets for interactions between virus and cell, such as the cell membrane or proteoglycans on the cell surface. Herein, bottom-up assembled synthetic SARS-CoV-2 miniviruses (MiniVs), with a lipid composition similar to that of infectious particles, are implemented to study and compare the binding properties of Omicron and Alpha variants. Toward this end, a systematic functional screening is performed to study the binding ability of Omicron and Alpha S proteins to ACE2-functionalized and nonfunctionalized planar supported lipid bilayers. Moreover, giant unilamellar vesicles are used as a cell membrane model to perform competitive interaction assays of the two variants. Finally, two cell lines with and without presentation of the ACE2 receptor are used to confirm the binding properties of the Omicron and Alpha MiniVs to the cellular membrane. Altogether, the results reveal a significantly higher affinity of Omicron S toward both the lipid membrane and ACE2 receptor. The research presented here highlights the advantages of creating and using bottom-up assembled SARS-CoV-2 viruses to understand the impact of changes in the affinity of S for ACE2 in infection studies.

## Introduction

Severe acute respiratory syndrome coronavirus 2 (SARS-CoV-2) is the causing agent of the still ongoing COVID-19 pandemic. Since the characterization of the viral strain in December 2019, thousands of variants have been identified and classified into five variants of concern named Alpha (B.1.1.7), Beta (B.1.351), Gamma (P.1), Delta (B.1.617.2), and Omicron (B.1.1.529).^1^ Each variant harbors different mutations, some of which lead to advantages in terms of immune system escape, transmissibility, or pathogenesis.^2^ SARS-CoV-2 infection starts with specific binding to the cell surface angiotensin-converting enzyme 2 (ACE2) which serves as a receptor, followed by fusion with the membrane or endosome and finally the release of the viral genetic material in the cytosol.^3^

The virus interacts with ACE2 through the spike glycoprotein (S), which coats the virion lipid bilayer and provides the virus with a crown-like appearance. The affinity of S for the receptor varies between variants, and this depends on the number and type of mutated amino acid residues. For example, the Omicron subvariants present up to 40 mutated amino acids compared to the original strain.^4^ The changes in the residue composition of the glycoprotein result in the appearance of complementary binding sites as well as modifications in its surface charge.^5^ The increase in positively charged amino acids in S, especially those in the receptor binding domain (RBD), indicate the possibility that the Omicron variant may have developed an alternative mechanism for infecting cells, a process that also relies on molecular interactions with the cell plasma membrane or polysaccharides located on the cell surface.^6^

Alsaadi et al. showed the ability of the Middle East respiratory syndrome-related coronavirus (MERS-CoV) S to bind to lipid membranes, although this study focused only on the S2 subunit of S.^7^ Moreover, it was shown that over time MERS-CoV S underwent viral adaptation by modifying the surface charge from negative to positive.^8^ Additionally, more recent studies have highlighted the importance of certain lipid species for the infectivity of SARS-CoV-2. For example, cholesterol alone and as a part of lipid rafts has been proposed to be essential for virus entry into the cell.^9^ However, these lipids were often considered as a recruitment platform for ACE2 and other receptors, not as interaction sites by themselves. Several studies have linked RBD mutations to increased viral infectivity. However, handling of SARS-CoV-2 under biosafety level 3 guidelines narrows the type of study that can be performed.^10^ In particular, purification and inactivation protocols are rarely compatible studies of viral biophysical properties. To overcome these and reduce the handling biosafety level, alternative strategies have been developed. Pseudoviruses appeared in the 1970s as a tool for host gene transfer from one cell to another.^11^ Since then, other synthetic top-down methods have been developed to study viruses in a more controlled manner, e.g. empty virus-like particles and quasivirions encapsulating full-length genomes.^12^ Specifically for SARS-CoV-2, both pseudoviruses and virus-like particles that superficially mimic the natural virus have been employed.^13,14^ Another approach has been to generate extracellular vesicles expressing SARS-CoV-2 S.^15^ Although all three listed systems result in safe models to study the properties of the virus, their production remains a challenge. Unlike MiniVs, the aforementioned top-down methods do not allow quantitative interaction and affinity measurements, neither easy composition changes in terms of lipid and protein content nor the ratio of S on the surface.

In this study, we take advantage of the tools provided by bottom-up synthetic biology for the controlled formation of SARS-CoV-2 viruses.^16^ We assembled synthetic SARS-CoV-2 MiniVs to study the change in the affinity of the Omicron and Alpha S variants for ACE2 and lipid membranes. Utilizing small unilamellar vesicles (SUVs) with a precise lipid composition—mimicking that of the natural SARS-CoV-2—as a backbone for functionalization with the S variant of interest, we formed synthetic MiniVs of use under BSL-1 conditions.^17^ We performed a systematic functional screening of binding of the Omicron and Alpha S to ACE2-functionalized and nonfunctionalized supported lipid bilayers (SLBs) using Quartz Crystal Microbalance with Dissipation monitoring (QCM-D). Moreover, we used giant unilamellar vesicles (GUVs) as a cell model and performed competitive interaction assays of the two S variants. To confirm the findings obtained with the QCM-D and GUV-based synthetic cells, two cell lines expressing different levels of ACE2 on their surface were ultimately employed. The results revealed a significantly higher affinity of the Omicron S for ACE2 compared to the Alpha S, as well as an interaction of the Omicron S with the lipid membrane. Our research highlights the advantages of implementing bottom-up assembled SARS-CoV-2 MiniVs as a tool to study S binding affinity in a well-controlled manner and identify additional interacting partners that will guide a future understanding of SARS-CoV-2 entry mechanisms.

## Results and Discussion

**Figure 1:**
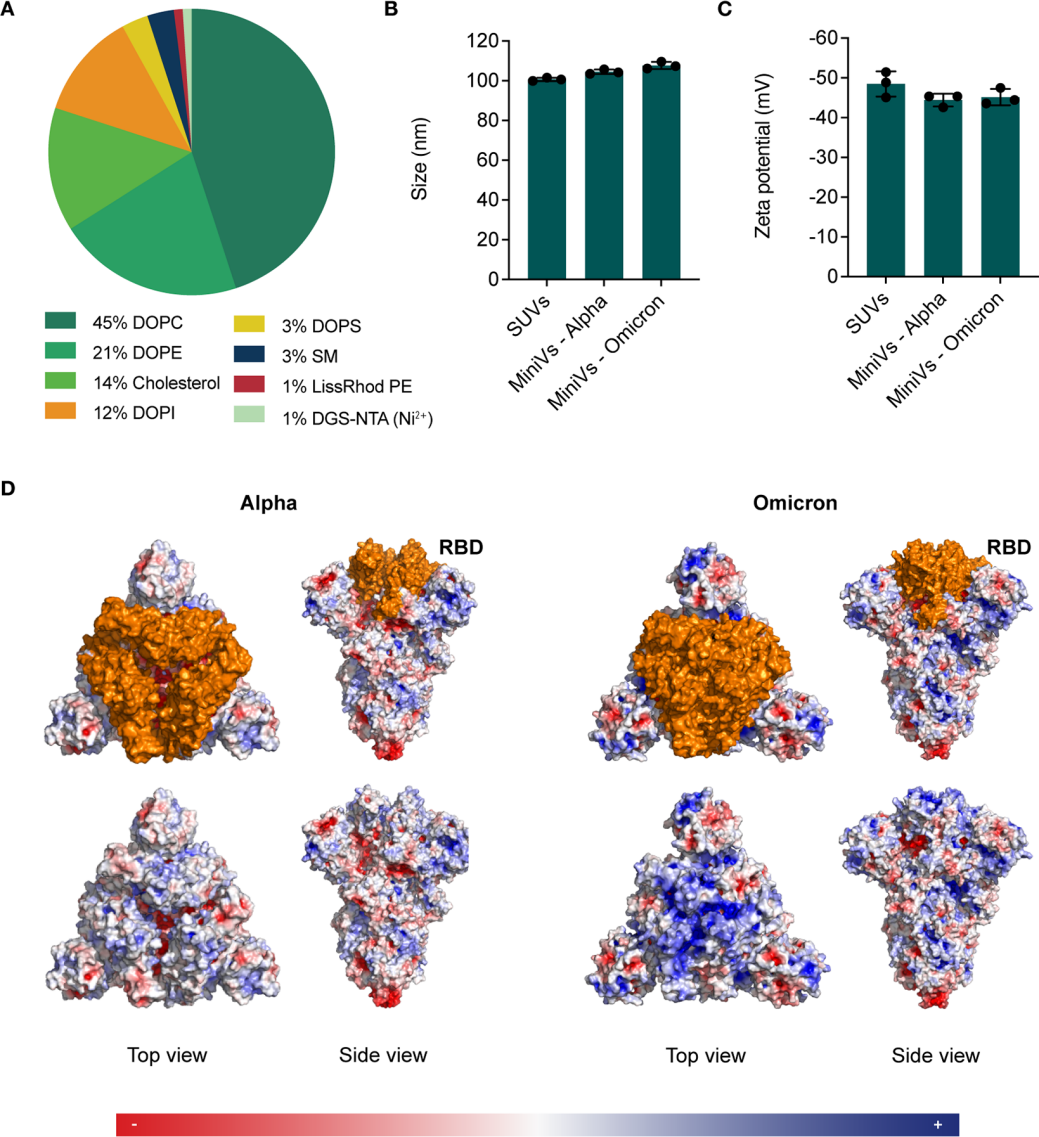
SUV and MiniV characterization. (A) SUV lipid composition. (B) SUVs and MiniVs size distribution analysis obtained by nanoparticle tracking analysis. (C) Zeta potential values of SUVs and MiniVs obtained by dynamic light scattering in Milli-Q water. Results in (B) and (C) correspond to the mean ± SD from *n* = 3 biological replicates under each experimental condition. (D) Surface charge of Alpha and Omicron S calculated by APBS and displayed in Pymol. To further improve the clarity of the presentation, the RBDs of Alpha and Omicron S (residues 319–541) are marked in orange.

The lipid composition employed for SUV formation (see Methods) was based on that of the endoplasmic-reticulum-Golgi intermediate compartment (ERGIC), a route used by SARS-CoV-2 for particle release (Figure 1A).^17^ Moreover, 1% of DGS-NTA(Ni^2+^) and 1% Liss Rhod PE lipids were included for biofunctionalization and labeling of the SUVs, respectively. Mass spectrometry was implemented to confirm the presence of all of the desired lipid species in the final vesicles (Figure S1A). MiniVs were obtained by biofunctionalization with either Omicron or Alpha histidine-tagged S via NTA(Ni^2+^)-functionalized lipids, reaching a size similar to that of natural SARS-CoV-2 virions of about 120 nm (Figure 1B).^18,19^ The stability of SUVs and MiniVs for a time period of 1 week was analyzed by measuring their size and concentration at different time points (Figure S2). Zeta potential measurements were performed to determine the surface charges of the SUVs and MiniVs in Milli-Q water. Due to their positively charged surface, MiniVs functionalized with Omicron S showed a lower negative charge than the SUVs and MiniVs functionalized with Alpha S (Figure 1C,D). Electrostatic potential surfaces of both S variants at pH 7.5 were visualized using the Adaptive Poisson–Boltzmann Solver (APBS) software and PyMol (The PyMOL Molecular Graphics System, Version 2.0 Schrödinger, LLC) (Figure 1D).^20^ In agreement with previous studies the surface of Omicron S (PDB 7WK2) showed a more positively charged surface specially around the receptor binding domain (RBD) in comparison to Alpha S (PDB 7LWS) (Figure 1D).^5^

**Figure 2:**
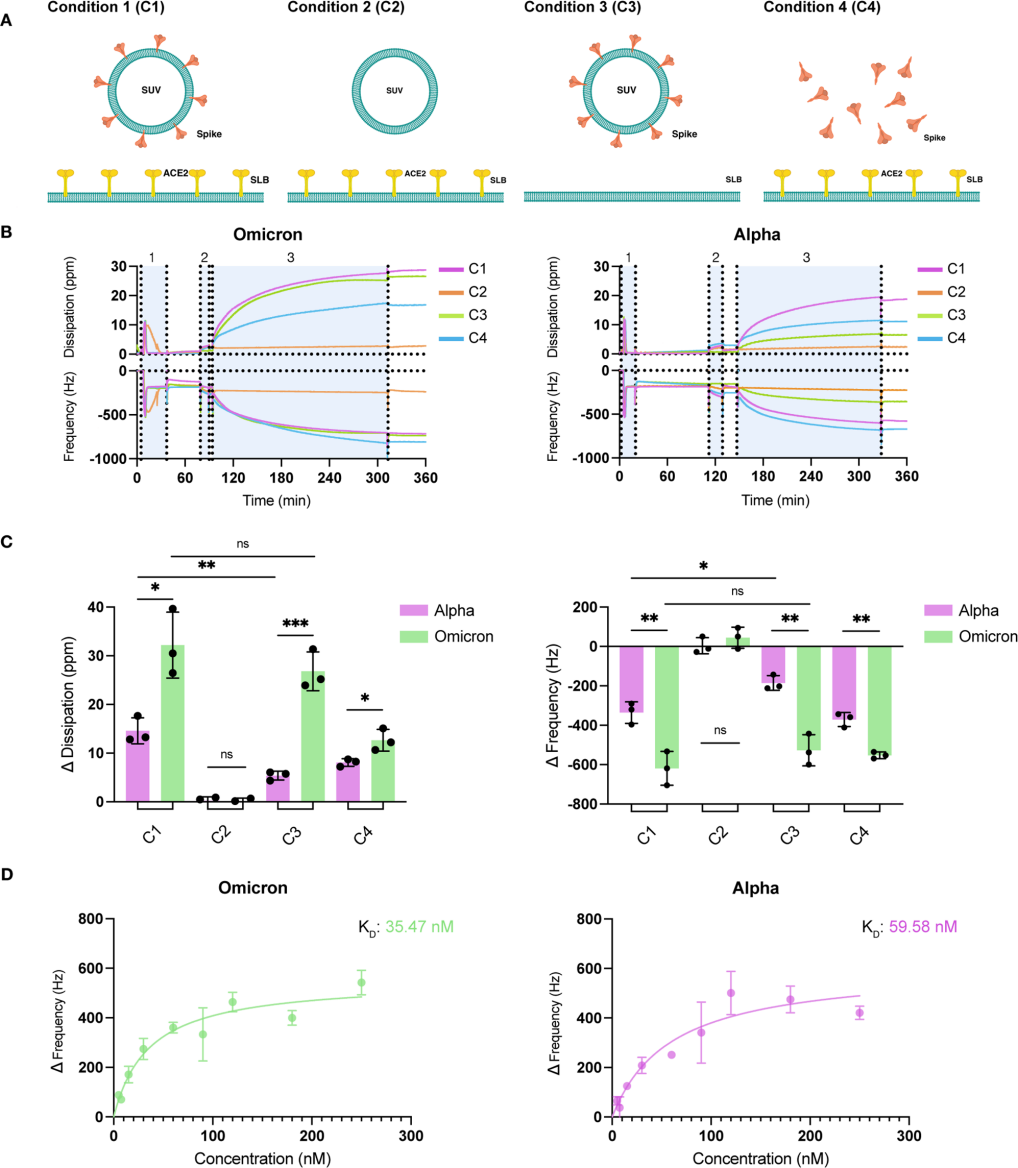
QCM-D experiments to determine Alpha and Omicron MiniV attachment to the ACE2-functionalized SLB. (A) Schematic illustration of the experimental conditions (C1–4) implemented in the experiments depicted in (B). Conditions 1 and 3 consist of Omicron/Alpha MiniVs interacting with an ACE2-functionalized or unfunctionalized SLB, respectively. Conditions 2 and 4 consist of nonfunctionalized SUVs or soluble S interacting with an ACE2-functionalized SLB, respectively. (B) Representative graphs depicting energy dissipation and frequency changes over time of Omicron (left) and Alpha (right) MiniVs. Phases marked in blue correspond to (1) SLB formation, (2) addition of ACE2, and (3) addition of MiniVs, SUVs, or soluble S. (C) Comparison of energy dissipation changes (left) and frequency changes (right) of Omicron and Alpha MiniVs. (D) Concentration curve of Omicron (left) or Alpha (right) S to determine the *K*_D_ of the interaction between the protein and the SLB. Results in (C) and (D) correspond to the mean ± SD from *n* = 3 biological replicates under each experimental condition, *p < 0.05, **p < 0.005, ***p < 0.001, analyzed using an unpaired two-tailed *t* test.

We used QCM-D to study the interaction of MiniVs with the ACE2 receptor and the SLB (see Methods).^21^ We compared the differences in SLB and ACE2 interaction between MiniVs, SUVs, and soluble S using different experimental conditions (Figure 2A). As can be observed in Figure 2B,C, Omicron MiniVs (C1) showed a significantly higher affinity toward the ACE2-functionalized SLB compared to nonfunctionalized SUVs (C2). Although Alpha MiniVs showed a higher affinity toward the ACE2 compared to SUVs, the affinity was significantly lower than that of Omicron MiniVs with ACE2 (Figure 2B,C). A higher affinity of Omicron S to ACE2 in comparison to other variants has been reported previously.^22^ Precisely, the dissociation constant (*K*_D_) of Omicron S binding to ACE2 has been documented as half that of Alpha S, with values of 5.5 and 11.8 nM, respectively.^23^

Recently it has been shown that the Omicron variant can bind to cells independently of the ACE2 receptor, via polysaccharides and proteoglycans.^6,5,24^ Although some molecular dynamics simulations have proven the interaction of Alpha and Omicron S with membranes, *in vitro* experiments confirming these results have yet to be reported.^25^ Interestingly, in contrast to Alpha MiniVs, Omicron MiniVs showed a similar affinity toward the unfunctionalized membrane (C3). To ensure that the changes in dissipation and frequency are S-driven, we performed an additional QCM-D experiment in which SUVs were in contact with a nonfunctionalized SLB (Figure S3). Quantitative analyses of energy dissipation and frequency change over time were not significantly different when comparing the interaction between Omicron MiniVs and either ACE2-functionalized (C1) or nonfunctionalized SLBs (C3) (Figure 2C). The obtained equilibrium dissociation constant (*K*_D_) between S and the SLB showed an almost 2-fold increase in affinity of Omicron S to the lipid membrane in comparison to Alpha S (Figure 2D and Figure S4). These results provide an indication of the affinity of the Omicron S binding to lipid membranes.

To test whether the planar geometry of the SLB affects the affinity of Omicron S, we decided to use an opposite experimental design and use ACE2-functionalized and nonfunctionalized SUVs and to test their affinity to Omicron and Alpha S-functionalized SLBs (Figure S5). It is important to mention that for these experiments, the lipid compositions of the SUVs and SLBs were the same as in the previous experiments presented here. The results of the opposite system confirmed the higher affinity of Omicron S toward ACE2-functionalized and nonfunctionalized lipid membranes. Together, these results suggest that changes in Omicron surface charge resulting from the accumulation of mutations in the RBD (Figure 1D) may have provided an advantage in efficiently binding lipid membranes in an ACE2-independent manner.

To better mimic the *in vivo* conditions, we decided to investigate and to compare the affinity of Omicron and Alpha MiniVs to the lipid membrane of GUVs whose lipid composition was similar to that of the SLB. To this end, we used the emulsion transfer method (see Methods) to produce either MiniVs- or SUVs-loaded GUVs or empty GUVs. In the case of empty GUVs, we added the MiniVs or SUVs to the solution surrounding the GUVs (hereinafter termed the external solution). GUVs were not labeled so as to avoid crosstalk between fluorophores. Thus, all recorded fluorescence resulted from SUVs or the MiniVs.

**Figure 3:**
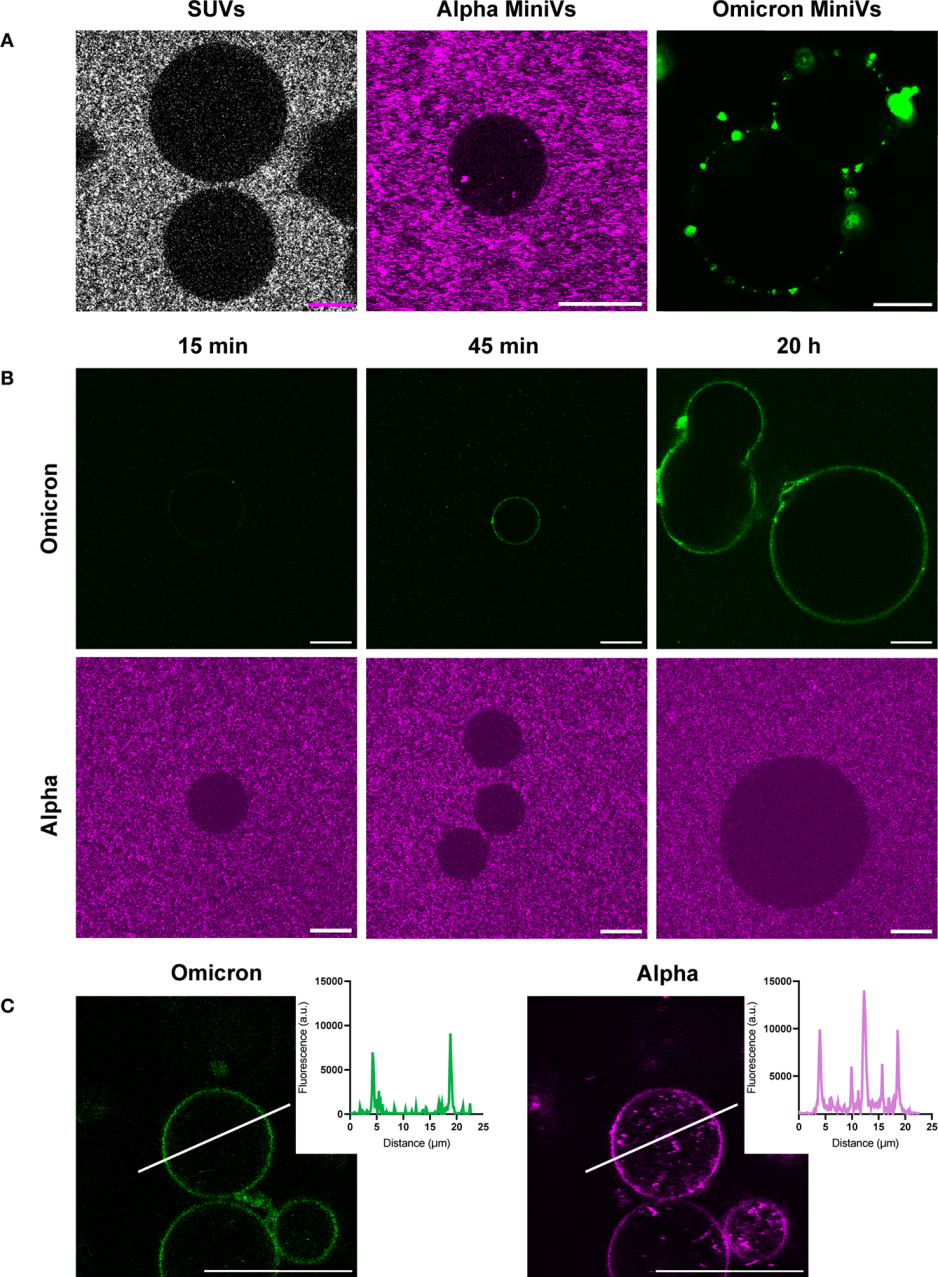
Representative confocal microscopy images of GUVs incubated with SUVs or MiniVs. (A) GUVs incubated with fluorescently labeled SUVs (left), Alpha MiniVs (middle), or Omicron MiniVs (right) (1% Liss Rhod PE). (B) Incubation over time of GUVs in contact with PEG-coated Omicron (top) or Alpha (bottom) MiniVs (1% Liss Rhod PE). For the sake of clarity, the colors in (A) and (B) are used with the only purpose of differentiating the conditions. (C) Competition assay to determine the affinity of the Omicron and Alpha MiniVs to the nonfunctionalized GUVs. The right insets show the intensity profile plots from the corresponding GUVs to their left. The fluorescence signals originate from the Omicron MiniVs (1% ATTO488 DOPE) (top) or Alpha MiniVs (1% ATTO647 DOPE) (bottom). All scale bars are 20 μm.

To recapitulate virus–cell interactions, MiniVs were added to the GUV solution. Omicron MiniVs (40 μM) added to the GUV external solution were able to interact with the outer vesicle membrane. This is in striking contrast to the behavior of Alpha MiniVs (40 μM), that similarly to unfunctionalized SUVs (40 μM) did not interact with the outer vesicle membrane (Figure 3A). Interestingly, the Omicron MiniVs that were added to the GUV external solution aggregated. This aggregation can be attributed to the charge-mediated self-interactions between the positively charged Omicron S proteins and the negatively charged MiniVs vesicles due to their prolonged exposure to each other before establishing an interaction with the GUVs. Note that the addition of ACE2 proteins to Omicron S functionalized MiniVs inhibited the aggregation, proving the involvement of Omicron S in MiniVs aggregation (Figure S6). To limit unspecific interactions between MiniVs and GUVs, MiniVs were produced with 4% PEG(2000)-functionalized lipids.^26,27^ The size and zeta potential measurements revealed that the addition of PEG-functionalized lipids to the vesicles did not lead to significant changes in charge or the dimensions of the obtained SUVs and Alpha or Omicron MiniVs (Figure S7). Despite the addition of PEG-functionalized lipids, the Omicron PEG-coated MiniVs established an interaction with the outer membrane of the GUVs that increased over time (Figure 3B). As expected, no interactions were observed between the Alpha PEG-coated MiniVs and GUVs (Figure 3B). These results confirm the affinity of Omicron S for the lipid membrane.

To assess the affinity of the Omicron and Alpha MiniVs to the lipid bilayer within the confinement, the SUVs or MiniVs were encapsulated inside the GUV lumen. Similar to the previous results, SUVs were randomly distributed within the lumen of the nonfunctionalized GUVs (Figure S8A). In contrast, both Alpha and Omicron MiniVs assembled along the membrane, forming a ring, indistinguishable from that formed in the presence of the ACE2 receptor on the GUV membrane (Figure S8B,C). However, the interaction of Alpha MiniV with the GUV membrane appeared to be weaker, as some of the signal from the Alpha MiniVs could be detected in the lumen of the GUV (Figure S8C). To determine which one of the S variants had a higher affinity toward the membrane, we performed a competition assay in which both Alpha and Omicron MiniVs of the same concentration were coencapsulated inside nonfunctionalized GUVs. As shown in the intensity profiles, Omicron MiniVs preferentially colocalized with the GUV membrane while Alpha MiniVs were randomly distributed within the GUV lumen (Figure 3C). In accordance with the QCM-D experiments, the affinity of the Omicron MiniVs for the lipid membrane was higher than that of the Alpha MiniVs.

In order to demonstrate the effect of surface charge in the binding of the Omicron S to lipid membranes, we produced positively charged GUVs and coencapsulated the Omicron and Alpha MiniVs. Due to the negative surface charge of Alpha S, Alpha MiniVs were able to efficiently interact with the positive GUV membrane, while Omicron MiniVs were distributed within the lumen (Figure S9).

To prove that the interaction with the GUV membrane is S-specific, we functionalized SUVs with ACE2 and encapsulated them inside GUVs (Figure S10). The ACE2-functionalized SUVs were randomly distributed within the lumen of the GUVs. Additionally, to exclude charge-mediated interactions of the MiniVs via NTA-functionalized lipids, we encapsulated Omicron MiniVs within GUVs that did not contain NTA-lipids. These MiniVs showed similarly strong interactions with the GUV membrane (Figure S11).

Even though commercially available S contains a nonfunctional fusion peptide, we sought to test whether Omicron S would be able to induce membrane fusion, due to its strong affinity to the lipid membrane. Therefore, a fusion assay was performed. Toward this end, either biotinylated SUVs or MiniVs were incubated with GUVs loaded with fluorescently labeled streptavidin. Under these conditions fusion of the MiniVs with the GUV would lead to the attraction of the encapsulated streptavidin to the biotinylated lipids, thereby causing the recruitment of streptavidin to the GUV membrane (Figure S12A). Despite their interactions, Omicron S MiniVs did not fuse with GUVs, as reflected by the random distribution of streptavidin molecules within the GUV lumen (Figure S12B).

**Figure 4:**
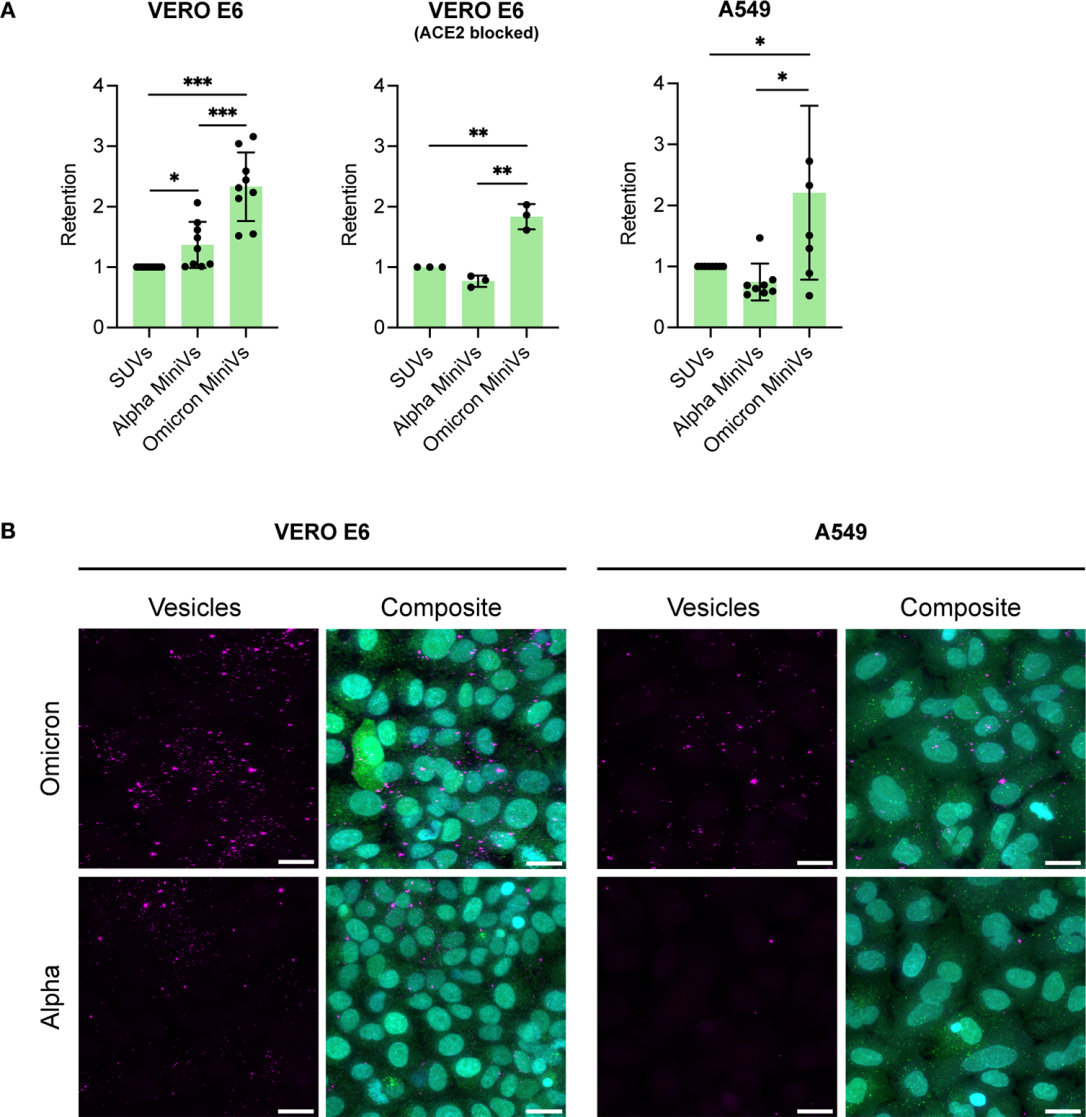
Flow cytometry screening of vesicle retention. (A) SUV, Alpha, and Omicron MiniV retention on Vero E6 and A549 cells screened by flow cytometry. (B) Representative images of maximal confocal microscopy *z*-projections of Vero E6 and A549 cells incubated for 1 h with Omicron and Alpha MiniVs (magenta). Cells were stained with CellTrackerGreen CMFDA (cytoplasm, green) and Hoechst 33342 (nucleus, cyan). The scale bars are 20 μm. Results in (A) correspond to the mean ± SD from at least *n* = 3 biological replicates under each experimental condition, *p< 0.05, **p< 0.005, ***p <0.001, analyzed using an unpaired two-tailed *t* test.

To compare the affinity of the Omicron and Alpha MiniVs for cellular membranes, Vero E6 cells expressing high levels of surface ACE2 and A549 cells lacking ACE2 expression (Figure S13) were used to perform retention assays. Flow cytometry measurements revealed a significantly higher retention of the Omicron MiniVs on the Vero E6 cell surface in comparison to Alpha MiniVs or SUVs (Figure 4A), in agreement with our QCM-D experiments. Inhibition of S binding to ACE2 by incubation of Vero E6 cells with an anti-ACE2 monoclonal antibody confirmed the added membrane affinity of Omicron MiniVs compared with Alpha MiniVs. This result is consistent with that obtained in A549 cells lacking ACE2 surface expression (Figure 4A). Confocal microscopy images of the cells incubated with SUVs and Omicron and Alpha MiniVs confirmed the results obtained with flow cytometry and showed an increased membrane retention of the Omicron MiniVs in both cell lines (Figure 4B and Figure S14).

## Conclusions

In this study, we implemented bottom-up synthetic biology for the controlled assembly of Alpha and Omicron SARS-CoV-2 MiniVs to study S-mediated affinity to lipid membranes. We have shown that the assembled MiniVs can be easily tuned in terms of the membrane and protein composition to fit the characteristics of the natural virus. The modularity of MiniVs provides the potential for future modifications to their design, enabling researchers to investigate the significance of lipid and protein composition in virus infectivity.

Using ACE2-functionalized and nonfunctionalized SLBs and GUVs, we assessed the effect of mutations in S reported in different variants of interest in its affinity to lipid membranes. We reported a high affinity of Omicron S to the lipid membrane in the absence of the ACE2 receptor. We further conducted a simple competition assay that allowed us to compare the interaction of MiniVs with functionalized and unfunctionalized synthetic lipid membranes. Ultimately, we screened the ability of Alpha and Omicron S to bind to a cell membrane lacking ACE2 receptors, which confirmed the results obtained in the QCM-D and in the GUV experiments.

Altogether, the outcomes of this study highlight the importance of the lipids present in the cell membrane to the SARS-CoV-2 Omicron infection process, which may participate in Omicron variants higher transmissibility.^28^ Better interaction and affinity with membranes might contribute to the broader cell tropism of the Omicron compared with previous variants. The discovered cell interaction sites of Omicron S might help to understand the variant’s lower viral replication competence in the lungs, but higher in the upper respiratory tract (e.g., nasal and bronchial tissue) compared to other variants.^29,30^ The findings in this paper emphasize the advantages of using bottom-up synthetic biology to study in a simple, quantitative, and tunable manner complex processes like virus–cell interactions and provide relevant information for subsequent studies with the natural virus.

## Methods

### Materials

1,2-Dioleoyl-*sn*-glycero-3-phosphoethanolamine-*N*-(lissamine rhodamine B sulfonyl) (ammonium salt) (18:1 Liss Rhod PE), cholesterol, 1,2-dioleoyl-*sn*-glycero-3-phosphocholine (18:1 (Δ9-Cis) PC, DOPC), 1,2-dioleoyl-*sn*-glycero-3-phosphoethanolamine (18:1 (Δ9-Cis) PE, DOPE), *N*-nervonoyl-*D*-erythro-sphingosylphosphorylcholine (24:1 SM), 1,2-dioleoyl-*sn*-glycero-3-phospho-*L*-serine (sodium salt) (18:1 PS), 1,2-dioleoyl-*sn*-glycero-3-[(*N*-(5-amino-1-carboxypentyl)iminodiacetic acid)succinyl] (18:1 DGS-NTA(Ni^2+^)), 1,2-dioleoyl-*sn*-glycero-3-phospho(1′-myo-inositol) (ammonium salt) (18:1 PI, DOPI), 1,2-dioleoyl-*sn*-glycero-3-phospho(1′-rac-glycerol) (sodium salt) (18:1 (Δ9-Cis) PG, DOPG), *L*-α-phosphatidylcholine (Egg PC), *L*-α-phosphatidylglycerol (Egg PG), 1,2-dioleoyl-*sn*-glycero-3-phosphoethanolamine-*N*-[methoxy(polyethylene glycol)-2000] (ammonium salt) (18:1 PEG2000 PE), and extrude set with 50 and 100 nm pore size polycarbonate filter membranes were purchased from Avanti Polar Lipids, USA. Dulbecco’s Modified Eagle Medium (DMEM) High Glucose, heat-inactivated fetal bovine serum, penicillin–streptomycin (10000 U/mL), Stempro Accutase Cell Dissociation Reagent, Nunc Lab-Tek Chamber Slide System, Hoechst 33342, CellTrackerGreen CMFDA, and Alexa-Fluor405 tagged streptavidin were purchased from Fischer Scientific, USA. OptiPrep Density Gradient Medium, silicone oil (viscosity 50 cSt), mineral oil, imidazole hydrochloride, Atto 647N DOPE, Atto 488 DOPE, cholesterol-d7, and EquiSPLASH were purchased from Merck, Germany. Human angiotensin-converting enzyme 2 (ACE2) protein (His Tag), SARS-CoV-2 B.1.1.529 (Omicron) and B.1.1.7 (Alpha) S1+S2 trimer Protein (ECD, His Tag), and Anti-ACE2 Antibody (FITC), Mouse Monoclonal (10108-MM37-F) were purchased from Sino Biological, USA. QCM-D sensor crystals (QS-QSX303) were purchased from Biolin Scientific, Sweden. FITC Mouse IgG1, κ Isotype Ctrl Antibody (400107) was purchased from Biolegend, USA.

### SUV/MiniV Preparation

SUVs were prepared by manual extrusion through track-etched polycarbonate filter membranes. To acquire the desired lipid ratio, stock lipids dissolved in chloroform were mixed in a glass vial at the desired lipid ratio following the composition previously described.^17^ Where needed, 4 mol % of 1,2-dioleoyl-*sn*-glycero-3-phosphocholine (DOPC) was substituted by 1,2-dioleoyl-*sn*-glycero-3-phosphoethanolamine-*N*-[methoxy(polyethylene glycol)-2000] (ammonium salt) (18:1 PEG2000 PE). The lipid mix was then dried under vacuum for at least 15 min. The obtained lipid film was rehydrated with filtered PBS to a final concentration of 6 mM for 5 min and then shaken at 1000 rpm for 5 min. The resulting polydisperse multilamellar vesicle population was then extruded through a 50 nm radius filter membrane to obtain the desired vesicle size.

To prepare MiniVs, the preformed SUVs were incubated with the histidine-tagged S for at least 45 min. The final protein concentration was one-sixth of the final molar concentration of DGS-NTA(Ni^2+^) lipid for QCM-D and GUV experiments.

### Mass Spectrometry

SUVs were prepared as described above. However, the membrane composition was adjusted to facilitate the MS measurements. Thus, the NTA-functionalized lipids were removed from the formulation, and the concentration (mol %) of cholesterol was doubled. Two sets of samples were generated comprising the unprocessed lipid solutions and redissolved free lipids after the SUV preparation process. All sample solutions were spiked with an internal standard (IS) mixture comprising Avanti’s EquiSPLASH LIPIDOMIX quantitative mass spec internal standard and cholesterol-d7. The addition of internal standards allows for the correction of variations due to sample preparation and acquisition as well as lipid specific nonlinear concentration-dependent effects.

Sample-IS mixtures were diluted in ACN to yield final concentrations of 3 μM for the total content of lipids and steroids (see Figure S1A for ratios), 0.3 μg mL^–1^ for cholesterol-d7, and 0.1 μg mL^–1^ for the total lipid content of the EquiSPLASH mixture. The resulting sample sets were produced and analyzed in triplicate.

The ratiometric analysis of lipid and steroid vesicle components was performed via LC-MS/MS. For this purpose, a Shimadzu Nexera UPLC (HILIC setup) hyphenated with a Sciex QTRAP 4500 triple-quadrupole mass spectrometer was used. The LC system was equipped with a Waters XBridge Amide column (3.5 μ, 4.6 mm × 150 mm), which was operated at 35 °C. 8 μL of the final sample solutions was injected on-column. A solvent system composed of solvent A (50% v/v ACN, 50% v/v H_2_O supplemented with 10 mM ammonia acetate, adjusted to pH 8) and solvent B (95% v/v ACN, 5% v/v H_2_O supplemented with 10 mM ammonia acetate) was used for compound elution. All solvents and additives were LCMS grade or higher. The respective parameters of the applied solvent concentration and flow gradients are supplied in Figure S1B. An increased flow rate of up to 1 mL min^–1^ was applied in the time period between 13 and 23 min to facilitate fast and efficient column cleaning and, hence, the prevention of lipid carryover. Solvent streams with elevated flow rate were diverted to the waste.

Data acquisition was performed via multi reaction monitoring (MRM) controlled by the Sciex Analyst 1.7.2 software. MSMS fragmentation patterns and resulting MRM parameters were determined via the infusion of respective standard solutions into 50% v/v dichloromethane and 50% v/v methanol supplemented with 10 mM ammonia acetate, thus allowing for adduct formation. The analyzed pre- and postprocess mixtures of vesicle components and internal standards contained compounds featuring significantly different ionizabilities due to their chemical composition. Accordingly, for cholesterol, which features only a single alcohol group attached to its aliphatic steroid structure, a drastically increased limit of detection was observed compared to e.g. DOPG bearing a quaternary and permanently charged amine. Nevertheless, a simultaneous analysis of all compounds of interest was possible due to a segmentation of the MS method. Segment 1 was fine-tuned for the detection of cholesterol by adjusting the source temperature to a lower temperature than segment 2. Changing the source temperature during the analysis is comparatively slow but was possible due to a sufficient retention time difference (>2.3 min) of cholesterol compared to the other lipidic vesicle components. Segment 2 was optimized for lipid analysis and featured polarity switching to account for the different ionizability of the various lipid head groups. Even though DOPC showed a better response in positive ionization mode, it was measured in negative ionization mode to prevent detector saturation in positive mode. Segment 3 restored the starting conditions of the MS method to allow for the subsequent measurement of additional samples. The detailed parameters of the MS method are provided in Figure S1C.

Initial data analysis was performed using the Sciex MultiQuant 3.0.2 software to provide the areas under the curve (AUC) for all analytes and their IS. Liss Rhod PE was not IS corrected. The coefficient of variations for IS corrected AUCs (triplicates) of cholesterol, DOPS, and DOPI were <15% and <5% for all other lipids. For subsequent data visualization, a Python script was used.^31−36^ The following formulas were used to calculate the percentage of each lipid species in the final SUV solution (Figure S1A). AUC_lipid_ and AUC_SUV_ correspond to the AUC values of each identified lipid species from the unprocessed lipid sample and the SUV sample, respectively. Percentage_lipids_ corresponds to the theoretical ratios of lipid species that were expected to be in the analyzed sample.
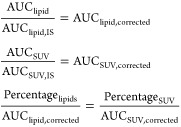


### Vesicle Characterization

Concentration, size, and zeta potential of SUVs and MiniVs was determined by nanoparticle tracking analysis with ZetaView QUATT (Particle Metrix, Inning am Ammersee, Germany). Alignment of the equipment was performed with 100 nm polystyrene beads diluted 1:250000 (v/v) in Milli-Q water. Vesicles were diluted in Milli-Q water to a final concentration of 60 nM. The observation cell was equilibrated with Milli-Q water (20 mL) before starting the analysis. For all experiments, nanoparticle tracking analysis was performed by scanning 11 positions of the observation cell. For size, zeta potential, and particle concentration measurements in water or PBS, the parameters were as follows: scatter mode (488 nm laser), temperature of 24 °C, minimal brightness 30, minimal area 10, maximal area 1000, trace length 15, sensitivity 80, frame rate 15, and shutter 100. For zeta potential measurements, the continuous mode was used due to the low conductivity of the medium (<2 mS cm^–1^). When DMEM was used as a dilution medium of the vesicles, sensitivity was reduced to 70.

### QCM-D Experiments

Sensor crystals coated with a layer of silicon oxide were used for the QCM-D measurements. The SiO_2_ surfaces were cleaned under UV light for 10 min and then placed in each module of the QCM-D analyzer in the open mode. A QSense Analyzer equipped with a four-channel system from QuantumDesign was used for measurements. The resonance frequency and energy dissipation shifts were recorded at several harmonics simultaneously. The four sensors were initially calibrated with PBS buffer for 5 min by adding 200 μL to each surface. To form the SLB, SUVs with a lipid composition of 78 mol % *L*-α-phosphatidylcholine (EggPC), 20 mol % *L*-α-phosphatidylglycerol (EggPG), and 2 mol % 1,2-dioleoyl-*sn*-glycero-3-[(*N*-(5-amino-1-carboxypentyl)iminodiacetic acid)succinyl] (18:1 DGS-NTA(Ni2+)) were diluted in PBS to a final lipid concentration of 1.2 mM and a final MgCl_2_ concentration of 2 mM. During the SUV formation process, the lipid film was rehydrated in 10 mM MgCl_2_ PBS and the resulting vesicle population was extruded through a 100 nm radius filter membrane. The SLB is formed by absorption and rupture of the SUVs on the activated SiO_2_ surfaces. 200 μL of the vesicle solution was added to each well and incubated for at least 30 min. Confirmation of the SLB formation was confirmed by characteristic changes in energy dissipation and frequency as described elsewhere.^37^ The lipid bilayers were washed with PBS to remove nonruptured vesicles. Histidine-tagged ACE2 protein was diluted in PBS to a final concentration of 300 nM, and 200 μL of the solution was added to the corresponding wells 1, 2, and 4. The solution was incubated for 15 min and then was replaced by PBS to remove unbound protein for 5 min. Next, 200 μL of a solution containing MiniVs to a final concentration of 40 μM was added to sensors 1 and 3. Sensor 2 was treated with 200 μL of 40 μM SUVs in PBS. A 200 μL portion of a PBS solution with 30 nM S was added to the surface of sensor 4. All wells were incubated with their respective vesicle or protein solution for a minimum of 2 h until a plateau in the energy dissipation and frequency curves was reached. Finally, all sensors were washed with PBS to remove unbound SUVs, MiniVs, or S and were left to equilibrate for at least 30 min.

An unpaired *t* test was performed to compare average values for energy dissipation and frequency using GraphPad Prism version 9.4.1 for Mac OS X. *K*_D_ values were determined by fitting the Hill equation assuming no cooperativity using GraphPad Prism version 9.4.1 for Mac OS X.

### GUV Preparation

GUVs were prepared using the emulsion transfer method, following previously described protocols.^38−40^ In short, a lipid-in-oil solution was prepared by mixing the desired phospholipids with mineral oil, 400 μL of which was placed on top of the outside aqueous phase. The remaining lipid-in-oil solution was mixed with the inner aqueous phase of the vesicle to form a water-in-oil emulsion. The emulsion was placed on the lipid monolayer formed on top of the outside aqueous phase, and final GUVs were obtained by centrifugation, removal of residual oil, and resuspension in adequate buffer. The phospholipid ratio used was 80 mol % 1,2-dioleoyl-*sn*-glycero-3-phosphocholine (DOPC) and 20 mol % 1,2-dioleoyl-*sn*-glycero-3-phospho-(1′-*rac*-glycerol) (sodium salt) (DOPG), substituting 2.5 mol % of DOPC with 18:1 DGS-NTA(Ni^2+^) where needed, achieving a final concentration of 643 μM in mineral oil (1 mL). The outside aqueous phase was composed of 5 mM imidazole-HCl buffer, and sucrose was added to match the osmolarity of the inner aqueous solution and Milli-Q water (in 500 μL). For empty GUVs, the inner aqueous phase was formed by 20% 5-[acetyl-[3-[acetyl-[3,5-bis(2,3- dihydroxypropylcarbamoyl)-2,4,6-triiodophenyl]amino]-2-hydroxypropyl]amino]-*N*,*N*′-bis(2,3-dihydroxypropyl)-2,4,6-triiodobenzene-1,3-dicarboxamide (OptiPrep) in PBS. For experiments with SUVs or MiniVs inside, the vesicles were added to the inner aqueous phase to a final concentration of 80 μM in PBS. ACE2 protein to a final concentration of 0.12 μM (for the inner buffer) or 0.3 μM (outside buffer) was added to the inner buffer when needed. In the MiniVs competition assay, PBS was used for the outside aqueous phase.

For positively charged GUVs, 20 mol % 1,2-dioleoyl-*sn*-glycero-3-phospho-(1′-rac-glycerol) (sodium salt) (DOPG) was substituted for 20 mol % 1,2-dioleoyl-3-trimethylammonium-propane (chloride salt) (DOTAP). Additionally, lipids were not diluted in mineral oil but in silicone oil (1 mL).

### Confocal Microscopy

All microscopy images were obtained with a LSM800 or LSM880 laser-scanning microscope (Carl Zeiss AG) using a 63× immersion oil objective (Plan-Apochromat ×63/1.40 Oil DIC, Carl Zeiss AG). All microscopy images shown in this paper were created using Fiji/ImageJ.^41^

### Cell Culture

A549 and Vero E6 cells were cultured in Dulbecco’s Modified Eagle Medium supplemented (DMEM) with 4.5 g/L glucose, 1% l-glutamine, 1% penicillin/streptomycin, and 10% fetal bovine serum. Cells were cultured at 37 °C and 5% CO_2_ atmosphere and were passaged at 90% confluency by detachment with Accutase.

### Flow Cytometry

Cells were detached with Accutase and centrifuged at 150 rcf for 5 min. The supernatant was discarded, and the cell pellet was resuspended in 2% PFA. After 10 min fixation, the cells were pelleted again by centrifuging at 150 rcf for 5 min. The cell pellet was resuspended in flow cytometry (FC) buffer (PBS 1% BSA 0.1% NaN_3_) to a final concentration of 5 × 10^5^ cells/mL and distributed in single eppis. For retention experiments, SUVs or Omicron or Alpha MiniVs to a final concentration of 7 μM were added separately to the cells and incubated for 1 h. For ACE2 quantification experiments, FITC-conjugated anti-ACE2 antibody or Mouse IgG1, κ Isotype antibody at a final concentration of 5 ng/mL was added to the cells and incubated in the dark for 1 h. For ACE2 inhibition experiments, before the addition of the vesicles, cells were incubated with the same antibody type and concentration described above. After 1 h incubation in the dark, the cells were centrifuged for 5 min at 4000 rcf to remove unbound antibody and resuspended in FC buffer.

Cells and vesicles were centrifuged for 5 min at 4000 rcf. The supernatant was removed, and the cells were resuspended in FC buffer and subsequently analyzed using a LSRFortessa instrument (Becton Dickinson). Analysis was performed using FlowJo software (Tree Star, Ashland, OR, USA).

An unpaired *t* test was performed to compare median fluorescence values of each condition using GraphPad Prism version 9.4.1 for Mac OS X.

### Cell Imaging

Cells were plated in 8-well chamber slides to a concentration of 5 × 10^5^ cells/mL and left overnight to form a monolayer. Cells were stained with Hoechst and CellTracker Green CMFDA for 40 min and then washed and fixed with 2% PFA for 10 min. SUVs or Alpha or Omicron MiniVs to a final concentration of 7 μM were added separately to the cells and incubated for 1 h. Cells were washed 3 times with PBS and imaged using confocal microscopy.

## Supporting Information Available

Additional experiment details and figures (PDF)
MS analysis: minimal working example how the mass spectrometry data was analyzed (ZIP)

## Abbreviations

SLB: supported lipid bilayer
GUV: giant unilamellar vesicle
SUV: small unilamellar vesicle
S: spike glycoprotein
ACE2: angiotensin-converting enzyme 2
QCM-D: quartz crystal microbalance with dissipation monitoring
NTA: nitrilotriacetic acid
PEG: polyethylene glycol
MiniVs: synthetic minimal virions
SARS-CoV-2: severe acute respiratory syndrome coronavirus 2
BSL: biosafety level
DOPC: 1,2-dioleoyl-*sn*-glycero-3-phosphocholine
DOPE: 1,2-dioleoyl-*sn*-glycero-3-phosphoethanolamine
DOPS: 1,2-dioleoyl-*sn*-glycero-3-phospho-*L*-serine
Liss Rhod PE: 1,2-dioleoyl-*sn*-glycero-3-phosphoethanolamine-*N*-(lissamine rhodamine B sulfonyl)
SM: 1*N*-nervonoyl-*D*-erythrosphingosylphosphorylcholine
DGS: NTA(Ni^2+^) 1,2-dioleoyl-*sn*-glycero-3-[(*N*-(5-amino-1-carboxypentyl)iminodiacetic acid)succinyl]
DOPI: 1,2-dioleoyl-*sn*-glycero-3-phospho(1′-myo-inositol)
DOPG: 1,2-dioleoyl-*sn*-glycero-3-phospho-(1′-rac-glycerol)

## References

[ref1] World Health Organization. Tracking SARS-CoV-2 variants. https://www.who.int/en/activities/tracking-SARS-CoV-2-variants/ (accessed 12-12-22).

[ref2] Natekar J. P.; Pathak H.; Stone S.; Kumari P.; Sharma S.; Auroni T. T.; Arora K.; Rothan H. A.; Kumar M. Differential Pathogenesis of SARS-CoV-2 Variants of Concern in Human ACE2-Expressing Mice. Viruses 2022, 14 (6), 1139 10.3390/v14061139. 35746611 PMC9231291

[ref3] Wang Q.; Zhang Y.; Wu L.; Niu S.; Song C.; Zhang Z.; Lu G.; Qiao C.; Hu Y.; Yuen K. Y.; Wang Q.; Zhou H.; Yan J.; Qi J. Structural and Functional Basis of SARS-CoV-2 Entry by Using Human ACE2. Cell 2020, 181 (4), 894–904. e9 10.1016/j.cell.2020.03.045. 32275855 PMC7144619

[ref4] Ou J.; Lan W.; Wu X.; Zhao T.; Duan B.; Yang P.; Ren Y.; Quan L.; Zhao W.; Seto D.; Chodosh J.; Luo Z.; Wu J.; Zhang Q. Tracking SARS-CoV-2 Omicron diverse spike gene mutations identifies multiple inter-variant recombination events. Signal Transduction and Targeted Therapy 2022, 7 (1), 138 10.1038/s41392-022-00992-2. 35474215 PMC9039610

[ref5] Nie C.; Sahoo A. K.; Netz R. R.; Herrmann A.; Ballauff M.; Haag R. Charge Matters: Mutations in Omicron Variant Favor Binding to Cells. Chembiochem 2022, 23 (6), e202100681 10.1002/cbic.202100681. 35020256 PMC9015620

[ref6] Gelbach A. L.; Zhang F.; Kwon S.-J.; Bates J. T.; Farmer A. P.; Dordick J. S.; Wang C.; Linhardt R. J. Interactions between heparin and SARS-CoV-2 spike glycoprotein RBD from omicron and other variants. Frontiers in Molecular Biosciences 2022, 9, 912887 10.3389/fmolb.2022.912887. 36046608 PMC9420978

[ref7] Alsaadi E. A. J.; Neuman B. W.; Jones I. M. A Fusion Peptide in the Spike Protein of MERS Coronavirus. Viruses 2019, 11 (9), 825 10.3390/v11090825. 31491938 PMC6784214

[ref8] Letko M.; Miazgowicz K.; McMinn R.; Seifert S. N.; Sola I.; Enjuanes L.; Carmody A.; van Doremalen N.; Munster V. Adaptive Evolution of MERS-CoV to Species Variation in DPP4. Cell Rep 2018, 24 (7), 1730–1737. 10.1016/j.celrep.2018.07.045. 30110630 PMC7104223

[ref9] Palacios-Rapalo S. N.; De Jesus-Gonzalez L. A.; Cordero-Rivera C. D.; Farfan-Morales C. N.; Osuna-Ramos J. F.; Martinez-Mier G.; Quistian-Galvan J.; Munoz-Perez A.; Bernal-Dolores V.; Del Angel R. M.; Reyes-Ruiz J. M. Cholesterol-Rich Lipid Rafts as Platforms for SARS-CoV-2 Entry. Front Immunol 2021, 12, 796855 10.3389/fimmu.2021.796855. 34975904 PMC8719300

[ref10] Syed A. M.; Ciling A.; Taha T. Y.; Chen I. P.; Khalid M. M.; Sreekumar B.; Chen P. Y.; Kumar G. R.; Suryawanshi R.; Silva I.; Milbes B.; Kojima N.; Hess V.; Shacreaw M.; Lopez L.; Brobeck M.; Turner F.; Spraggon L.; Tabata T.; Ott M.; Doudna J. A. Omicron mutations enhance infectivity and reduce antibody neutralization of SARS-CoV-2 virus-like particles. Proc. Natl. Acad. Sci. U. S. A. 2022, 119 (31), e2200592119 10.1073/pnas.2200592119. 35858386 PMC9351483

[ref11] Trilling D. M.; Axelrod D. Encapsidation of free host DNA by simian virus 40: a simian virus 40 pseudovirus. Science 1970, 168 (3928), 268–71. 10.1126/science.168.3928.268. 4313909

[ref12] Conway M. J.; Meyers C. Replication and assembly of human papillomaviruses. J. Dent Res. 2009, 88 (4), 307–17. 10.1177/0022034509333446. 19407149 PMC3317948

[ref13] Muik A.; Wallisch A. K.; Sanger B.; Swanson K. A.; Muhl J.; Chen W.; Cai H.; Maurus D.; Sarkar R.; Tureci O.; Dormitzer P. R.; Sahin U. Neutralization of SARS-CoV-2 lineage B.1.1.7 pseudovirus by BNT162b2 vaccine-elicited human sera. Science 2021, 371 (6534), 1152–1153. 10.1126/science.abg6105. 33514629 PMC7971771

[ref14] Xu R.; Shi M.; Li J.; Song P.; Li N. Construction of SARS-CoV-2 Virus-Like Particles by Mammalian Expression System. Front Bioeng Biotechnol 2020, 8, 862 10.3389/fbioe.2020.00862. 32850726 PMC7409377

[ref15] Verta R.; Grange C.; Skovronova R.; Tanzi A.; Peruzzi L.; Deregibus M. C.; Camussi G.; Bussolati B. Generation of Spike-Extracellular Vesicles (S-EVs) as a Tool to Mimic SARS-CoV-2 Interaction with Host Cells. Cells 2022, 11 (1), 146 10.3390/cells11010146. 35011708 PMC8750506

[ref16] Staufer O.; Gantner G.; Platzman I.; Tanner K.; Berger I.; Spatz J. P. Bottom-up assembly of viral replication cycles. Nat. Commun. 2022, 13 (1), 6530 10.1038/s41467-022-33661-7. 36323671 PMC9628313

[ref17] Staufer O.; Gupta K.; Hernandez Bucher J. E.; Kohler F.; Sigl C.; Singh G.; Vasileiou K.; Yague Relimpio A.; Macher M.; Fabritz S.; Dietz H.; Cavalcanti Adam E. A.; Schaffitzel C.; Ruggieri A.; Platzman I.; Berger I.; Spatz J. P. Synthetic virions reveal fatty acid-coupled adaptive immunogenicity of SARS-CoV-2 spike glycoprotein. Nat. Commun. 2022, 13 (1), 868 10.1038/s41467-022-28446-x. 35165285 PMC8844029

[ref18] Klein S.; Cortese M.; Winter S. L.; Wachsmuth-Melm M.; Neufeldt C. J.; Cerikan B.; Stanifer M. L.; Boulant S.; Bartenschlager R.; Chlanda P. SARS-CoV-2 structure and replication characterized by in situ cryo-electron tomography. Nat. Commun. 2020, 11 (1), 5885 10.1038/s41467-020-19619-7. 33208793 PMC7676268

[ref19] Zhu N.; Zhang D.; Wang W.; Li X.; Yang B.; Song J.; Zhao X.; Huang B.; Shi W.; Lu R.; Niu P.; Zhan F.; Ma X.; Wang D.; Xu W.; Wu G.; Gao G. F.; Tan W.; China Novel Coronavirus I.; Research T. A Novel Coronavirus from Patients with Pneumonia in China, 2019. N Engl J. Med. 2020, 382 (8), 727–733. 10.1056/NEJMoa2001017. 31978945 PMC7092803

[ref20] Jurrus E.; Engel D.; Star K.; Monson K.; Brandi J.; Felberg L. E.; Brookes D. H.; Wilson L.; Chen J.; Liles K.; Chun M.; Li P.; Gohara D. W.; Dolinsky T.; Konecny R.; Koes D. R.; Nielsen J. E.; Head-Gordon T.; Geng W.; Krasny R.; Wei G. W.; Holst M. J.; McCammon J. A.; Baker N. A. Improvements to the APBS biomolecular solvation software suite. Protein Sci. 2018, 27 (1), 112–128. 10.1002/pro.3280. 28836357 PMC5734301

[ref21] Rodahl M.; Höök F.; Krozer A.; Brzezinski P.; Kasemo B. Quartz crystal microbalance setup for frequency and Q-factor measurements in gaseous and liquid environments. Rev. Sci. Instrum. 1995, 66 (7), 3924–3930. 10.1063/1.1145396.

[ref22] Li L.; Liao H.; Meng Y.; Li W.; Han P.; Liu K.; Wang Q.; Li D.; Zhang Y.; Wang L.; Fan Z.; Zhang Y.; Wang Q.; Zhao X.; Sun Y.; Huang N.; Qi J.; Gao G. F. Structural basis of human ACE2 higher binding affinity to currently circulating Omicron SARS-CoV-2 sub-variants BA.2 and BA.1.1. Cell 2022, 185 (16), 2952–2960. e10 10.1016/j.cell.2022.06.023. 35809570 PMC9212699

[ref23] Kim S.; Liu Y.; Ziarnik M.; Cao Y.; Zhang X. F.; Im W. Binding of Human ACE2 and RBD of Omicron Enhanced by Unique Interaction Patterns Among SARS-CoV-2 Variants of Concern. J Comput Chem 2023, 44, 594 10.1002/jcc.27025. 36398990 PMC9825653

[ref24] Kim S. H.; Kearns F. L.; Rosenfeld M. A.; Votapka L.; Casalino L.; Papanikolas M.; Amaro R. E.; Freeman R., Positively bound: Remapping of Increased Positive Charge Drives SARS-CoV-2 Spike Evolution to Optimize its Binding to Cell Surface Receptors. ChemRxiv 2022 (accessed 1-26-23).

[ref25] Overduin M.; Kervin T. A.; Tran A. Progressive membrane-binding mechanism of SARS-CoV-2 variant spike proteins. iScience 2022, 25 (8), 104722 10.1016/j.isci.2022.104722. 35813872 PMC9251956

[ref26] Charles P. T.; Stubbs V. R.; Soto C. M.; Martin B. D.; White B. J.; Taitt C. R. Reduction of Non-Specific Protein Adsorption Using Poly(ethylene) Glycol (PEG) Modified Polyacrylate Hydrogels In Immunoassays for Staphylococcal Enterotoxin B Detection. Sensors (Basel) 2009, 9 (1), 645–55. 10.3390/s90100645. 22389622 PMC3280768

[ref27] Xu F. J.; Li H. Z.; Li J.; Teo Y. H.; Zhu C. X.; Kang E. T.; Neoh K. G. Spatially well-defined binary brushes of poly(ethylene glycol)s for micropatterning of active proteins on anti-fouling surfaces. Biosens Bioelectron 2008, 24 (4), 773 10.1016/j.bios.2008.06.055. 18684612

[ref28] Suzuki R.; Yamasoba D.; Kimura I.; Wang L.; Kishimoto M.; Ito J.; Morioka Y.; Nao N.; Nasser H.; Uriu K.; Kosugi Y.; Tsuda M.; Orba Y.; Sasaki M.; Shimizu R.; Kawabata R.; Yoshimatsu K.; Asakura H.; Nagashima M.; Sadamasu K.; Yoshimura K.; Genotype to Phenotype Japan C.; Sawa H.; Ikeda T.; Irie T.; Matsuno K.; Tanaka S.; Fukuhara T.; Sato K.; et al. Attenuated fusogenicity and pathogenicity of SARS-CoV-2 Omicron variant. Nature 2022, 603 (7902), 700–705. 10.1038/s41586-022-04462-1. 35104835 PMC8942852

[ref29] Hui K. P. Y.; Ho J. C. W.; Cheung M. C.; Ng K. C.; Ching R. H. H.; Lai K. L.; Kam T. T.; Gu H.; Sit K. Y.; Hsin M. K. Y.; Au T. W. K.; Poon L. L. M.; Peiris M.; Nicholls J. M.; Chan M. C. W. SARS-CoV-2 Omicron variant replication in human bronchus and lung ex vivo. Nature 2022, 603 (7902), 715–720. 10.1038/s41586-022-04479-6. 35104836

[ref30] Hui K. P. Y.; Ng K. C.; Ho J. C. W.; Yeung H. W.; Ching R. H. H.; Gu H.; Chung J. C. K.; Chow V. L. Y.; Sit K. Y.; Hsin M. K. Y.; Au T. W. K.; Poon L. L. M.; Peiris M.; Nicholls J. M.; Chan M. C. W. Replication of SARS-CoV-2 Omicron BA.2 variant in ex vivo cultures of the human upper and lower respiratory tract. EBioMedicine 2022, 83, 104232 10.1016/j.ebiom.2022.104232. 35988466 PMC9387351

[ref31] Rossum G. V.; Drake F. L. Python 3 Reference Manual: CreateSpace: 2009; p 242.

[ref32] Waskom M. seaborn: statistical data visualization. Journal of Open Source Software 2021, 6, 3021 10.21105/joss.03021.

[ref33] McKinney W. In Data Structures for Statistical Computing in Python. Scipy 2010.

[ref34] Hunter J. D. Matplotlib: A 2D Graphics Environment. Computing in Science & Engineering 2007, 9 (3), 90–95. 10.1109/MCSE.2007.55.

[ref35] Harris C. R.; Millman K. J.; van der Walt S. J.; Gommers R.; Virtanen P.; Cournapeau D.; Wieser E.; Taylor J.; Berg S.; Smith N. J.; Kern R.; Picus M.; Hoyer S.; van Kerkwijk M. H.; Brett M.; Haldane A.; Del Rio J. F.; Wiebe M.; Peterson P.; Gerard-Marchant P.; Sheppard K.; Reddy T.; Weckesser W.; Abbasi H.; Gohlke C.; Oliphant T. E. Array programming with NumPy. Nature 2020, 585 (7825), 357–362. 10.1038/s41586-020-2649-2. 32939066 PMC7759461

[ref36] Kluyver T.; Ragan-Kelley B.; Pérez F.; Granger B.; Bussonnier M.; Frederic J.; Kelley K.; Hamrick J.; Grout J.; Corlay S.; Ivanov P.; Avila D.; Abdalla S.; Willing C.; Team J. D. Jupyter Notebooks—a publishing format for reproducible computational workflows; IOS Press: 2016; pp 87–90.

[ref37] Briand E.; Zäch M.; Svedhem S.; Kasemo B.; Petronis S. Combined QCM-D and EIS study of supported lipid bilayer formation and interaction with pore-forming peptides. Analyst 2010, 135 (2), 343–50. 10.1039/B918288H. 20098769

[ref38] Fink A.; Doll C. R.; Yague Relimpio A.; Dreher Y.; Spatz J. P.; Gopfrich K.; Cavalcanti-Adam E. A. Extracellular Cues Govern Shape and Cytoskeletal Organization in Giant Unilamellar Lipid Vesicles. ACS Synth. Biol. 2023, 12 (2), 369–374. 10.1021/acssynbio.2c00516. 36652603 PMC9942188

[ref39] Ganzinger K. A.; Merino-Salomon A.; Garcia-Soriano D. A.; Butterfield A. N.; Litschel T.; Siedler F.; Schwille P. FtsZ Reorganization Facilitates Deformation of Giant Vesicles in Microfluidic Traps*. Angew. Chem., Int. Ed. Engl. 2020, 59 (48), 21372–21376. 10.1002/anie.202001928. 32735732 PMC7756778

[ref40] Moga A.; Yandrapalli N.; Dimova R.; Robinson T. Optimization of the Inverted Emulsion Method for High-Yield Production of Biomimetic Giant Unilamellar Vesicles. Chembiochem 2019, 20 (20), 2674–2682. 10.1002/cbic.201900529. 31529570 PMC6856842

[ref41] Schindelin J.; Arganda-Carreras I.; Frise E.; Kaynig V.; Longair M.; Pietzsch T.; Preibisch S.; Rueden C.; Saalfeld S.; Schmid B.; Tinevez J. Y.; White D. J.; Hartenstein V.; Eliceiri K.; Tomancak P.; Cardona A. Fiji: an open-source platform for biological-image analysis. Nat. Methods 2012, 9 (7), 676–82. 10.1038/nmeth.2019. 22743772 PMC3855844

