# Supplementary material for: Bottom-up Assembled Synthetic SARS-CoV-2 Miniviruses Reveal Lipid Membrane Affinity of Omicron Variant Spike Glycoprotein
Source: ACS Nano. 2023 Nov 17;17(23):23913–23. doi: 10.1021/acsnano.3c08323 (PMC10722588; doi:10.1021/acsnano.3c08323)
Supplement: Supplementary file 1 — nn3c08323_si_001.pdf [file nn3c08323_si_001.pdf]

Bottom-up assembled synthetic SARS-CoV-2 mini-viruses reveal lipid membrane affinity of Omicron variant spike glycoprotein

Ana Yagüe Relimpio[1,2], Andreas Fink[1], Duc Thien Bui[1], Sebastian Fabritz[3], Martin Schröter[1], Alessia Ruggieri[4], Ilia Platzman[1,2,5] and Joachim P. Spatz[1,2,5,6] **

[1]Department for Cellular Biophysics, Max Planck Institute for Medical Research, Jahnstrasse 29, 69120, Heidelberg, Germany

[2]Institute for Molecular Systems Engineering and Advanced Materials (IMSE), Heidelberg University, Im Neuenheimer Feld 225, 69120, Heidelberg, Germany

[3]Department for Chemical Biology, Max Planck Institute for Medical Research, Jahnstrasse 29, 69120, Heidelberg, Germany

[4]Heidelberg University, Medical Faculty, Centre for Integrative Infectious Disease Research (CIID), Department of Infectious Diseases, Molecular Virology, Im Neuenheimer Feld 344, 69120, Heidelberg, Germany

[5]Max Planck-Bristol Center for Minimal Biology, University of Bristol, 1 Tankard's Close, Bristol, BS8 1TD, UK

[6]Max Planck School Matter to Life, Jahnstrasse 29, 69120, Heidelberg, Germany

Supporting information

A

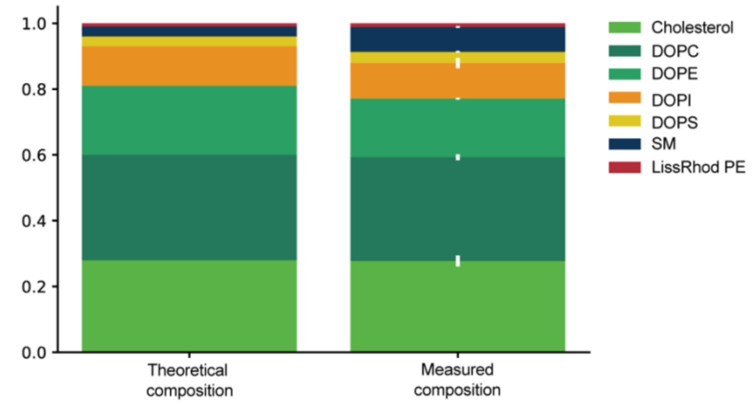

B

Time (min)	Parameter	
0.01	Solvent B %	99.90%
1.5	Solvent B %	99.90%
4	Solvent B %	94%
10.5	Solvent B %	40%
11.5	Solvent B %	0%
13	Total flow	0.55 mL
13.5	Solvent B %	0%
13.5	Total flow	1 mL
19.1	Solvent B %	0%
19.2	Solvent B %	99.90%
22.5	Total flow	1 mL
23	Total flow	0.55 mL
24.5	Stop	-

C

Experiment 1 (POS)

**Segment 1**	Curtain gas (CUR)	35	ID	Chol-NH4.1	Chol-H2O.2	Chol-NH4.IS-d7		
	Collision gas (CAD)	9	Q1 (Da)	404.283	369.400	411.280		
	Source temperature (TEM)	210	Q3 (Da)	369.400	147.300	376.400		
	Nebulizer gas (GS1)	65	Dwell	120.000	50.000	100.000		
	Heater gas (GS2)	70	DP	116.000	220.000	116.000		
	Ionization voltage (IS)	5500	CE	15.000	29.000	15.000		
	Entrance Potential (EP)	10	CXP	11.500	10.000	11.500		
**Segment 2**	Curtain gas (CUR)	35	ID	LissRhod	SM24	SM-d9	PE	PE-d7
from 3.6 min	Collision gas (CAD)	9	Q1 (Da)	1301.605	813.664	738.500	744.498	711.400
	Source temperature (TEM)	350	Q3 (Da)	682.000	184.100	184.000	603.500	570.500
	Nebulizer gas (GS1)	65	Dwell	30.000	30.000	25.000	25.000	25.000
	Heater gas (GS2)	70	DP	40.000	120.000	130.000	91.000	120.000
	Ionization voltage (IS)	5500	CE	67.000	37.000	37.000	33.000	30.000
	Entrance Potential (EP)	10	CXP	24.000	18.000	16.000	16.000	12.000
**Segement 3**	Curtain gas (CUR)	35	ID	Chol-NH4.1	Chol-H2O.2	Chol-NH4.IS-d7		
from 18.6 min	Collision gas (CAD)	9	Q1 (Da)	404.283	369.400	411.280		
	Source temperature (TEM)	210	Q3 (Da)	369.400	147.300	376.400		
	Nebulizer gas (GS1)	65	Dwell	120.000	50.000	100.000		
	Heater gas (GS2)	70	DP	116.000	220.000	116.000		
	Ionization voltage (IS)	5500	CE	15.000	29.000	15.000		
	Entrance Potential (EP)	10	CXP	11.500	10.000	11.500		

Experiment 2 (NEG)

Curtain gas (CUR)	35	ID	PC-OAc	PC-d7-OAc	PS	PS-d7	PI	PI-d7
Collision gas (CAD)	9	Q1 (Da)	844.5	811.5	786.5	753.4	861.413	828.4
Source temperature (TEM)	350	Q3 (Da)	281.3	288.3	281.3	288.3	281.3	288.3
Nebulizer gas (GS1)	65	Dwell	25	25	30	25	25	25
Heater gas (GS2)	70	DP	-90	-115	-120	-100	-185	-90
Ionization voltage (IS)	-4500	CE	-54	-50	-55	-56	-60	-60
Entrance Potential (EP)	-10	CXP	-7	-11	-8.5	-10	-11	-11

Figure S1. Lipid composition as measured by mass spectrometry. A) Lipid composition of re-dissolved SUVs determined via LC-MS/MS. Data in A corresponds to n = 3 biological replicates. B) UPLC gradient information. C) Source and MS parameters of the applied segmented LC-MS/MS (MRM) method. Segment 1 allows the measurement of cholesterol at low temperature and with high dwell times (ms). Segment 2 features an increased temperature and polarity switching with regard to the ionization process for the measurement of lipids. The parameters for

lipid fragment transitions measured in positive mode are supplied in the column labelled Experiment 1 (POS). The parameters for lipid fragment transitions measured in negative ionization mode are given in the column labelled Experiment 2 (NEG).

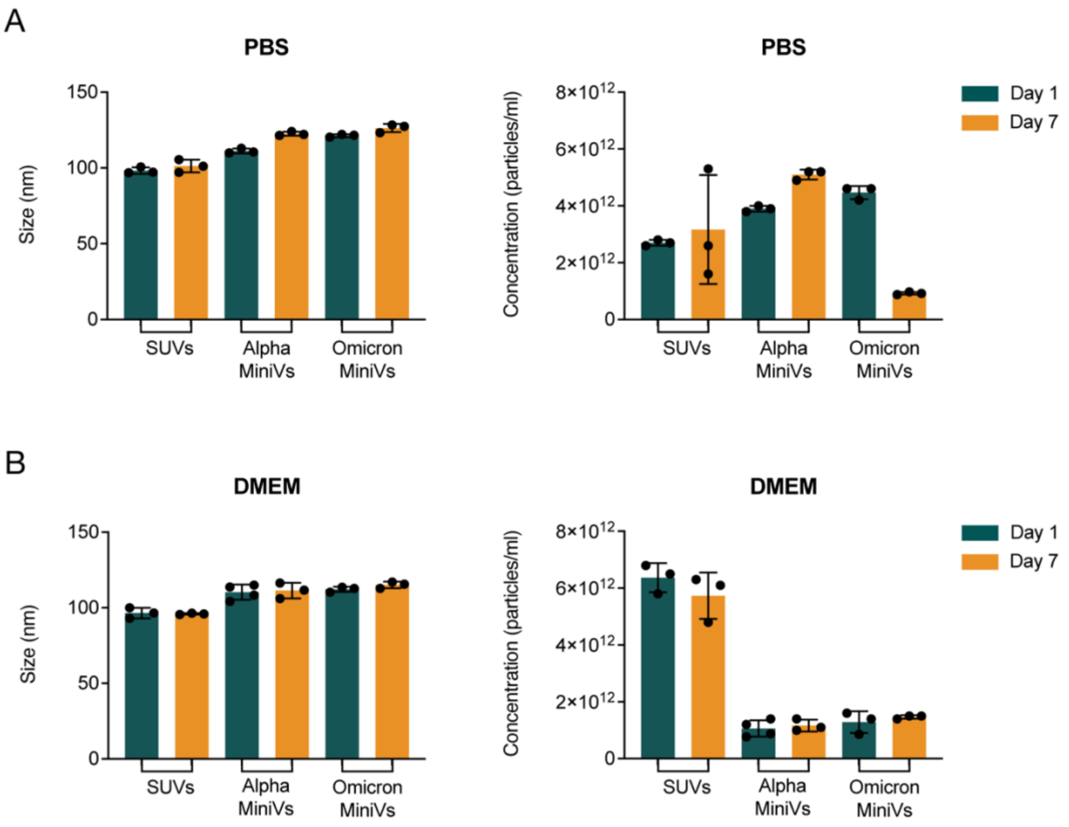

Figure S2. SUVs and MiniVs stability analysis over time obtained by nanoparticle tracking analysis. Size and concentration analysis of SUVs, Alpha and Omicron MiniVs after 1h and 7 days of incubation in PBS (A) and DMEM (B). Results correspond to the mean ± SD from n = 3 biological replicates in each experimental condition.

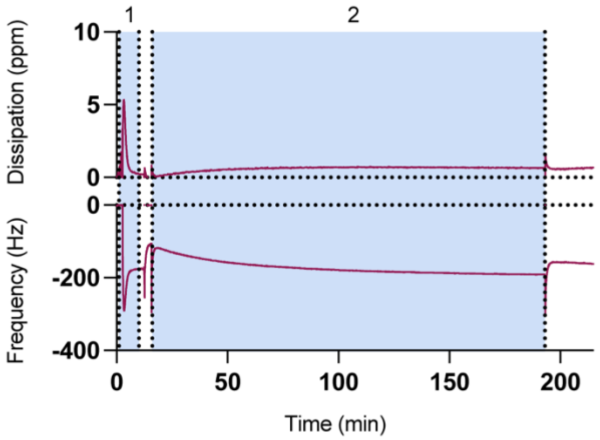

Figure S3. Over time energy dissipation and frequency change of SUVs in contact with a non-functionalized SLB. Phases marked in blue correspond to: 1) SLB formation; 2) Addition of SUVs.

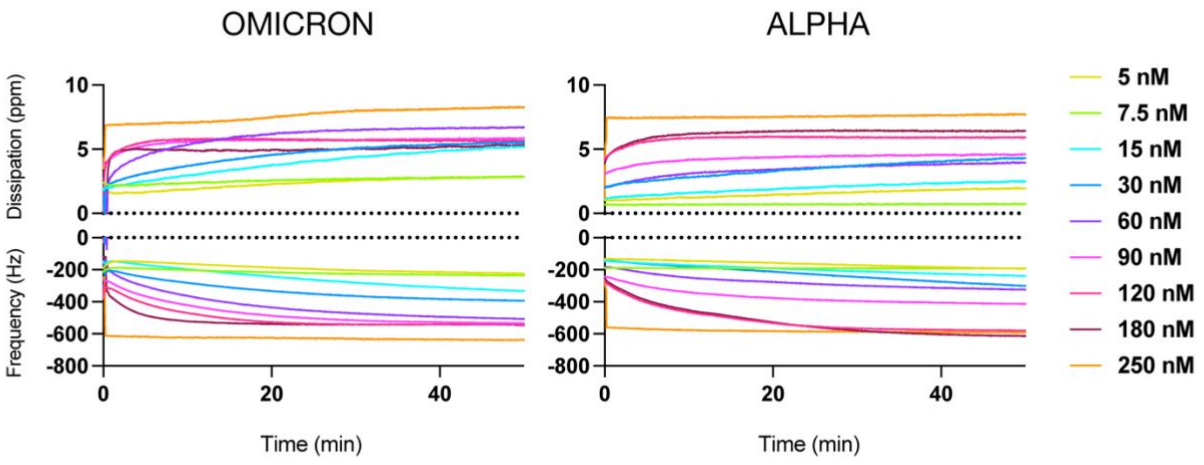

Figure S4. Over time energy dissipation and frequency change of Omicron (left) and Alpha (right) S in contact with a non-functionalized SLB.

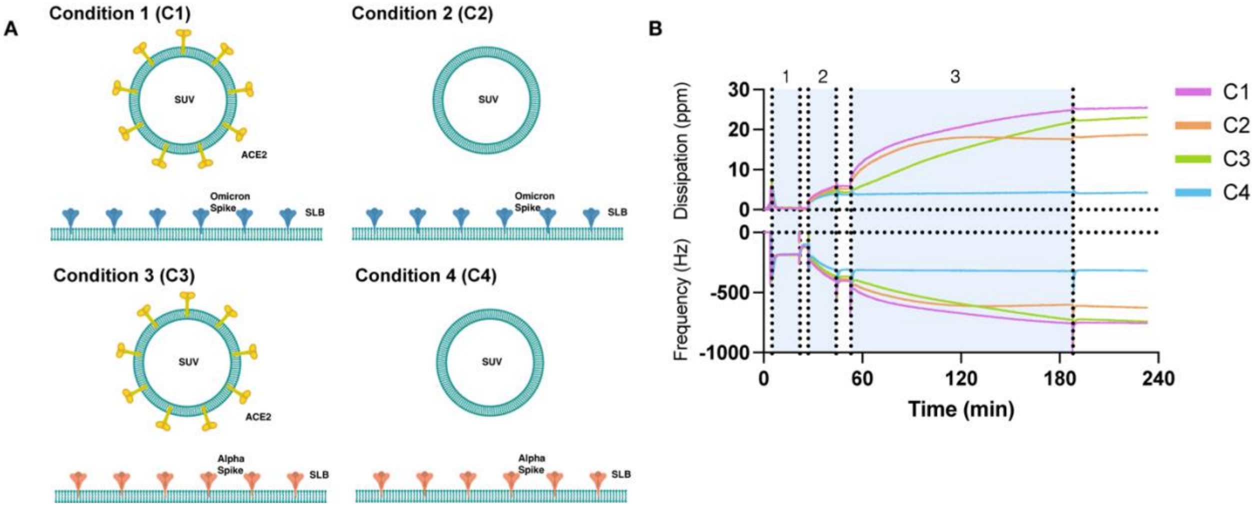

Figure S5. QCM-D measurement of Omicron or Alpha-functionalized SLBs. A) Schematic illustration of the experimental conditions (C1-4) implemented in the experiments depicted in B. Conditions 1 and 3 consist of Omicron or Alpha-functionalized SLBs in contact with ACE2-functionalized SUVs, respectively. Conditions 2 and 4 consist of Omicron or Alpha-functionalized SLBs in contact with naive SUVs, respectively. B) Representative graph depicting energy dissipation and frequency changes over time under the conditions defined in A). Phases marked in blue correspond to: 1) SLB formation, 2) Addition of Alpha S or Omicron S and 3) Addition of ACE2-functionalized SUVs or naive SUVs.

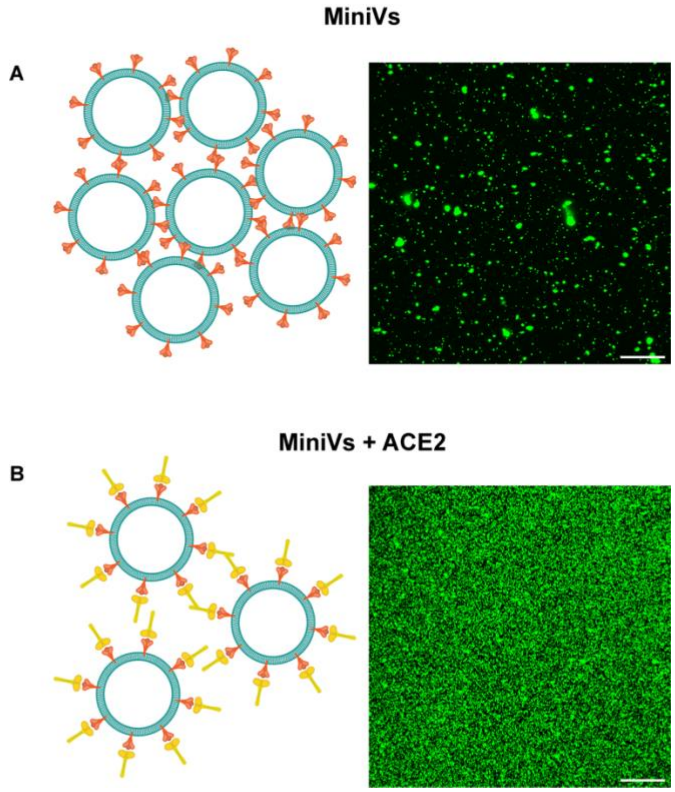

Figure S6. A) Schematic illustration of S-mediated Omicron MiniV aggregation (left) and representative confocal image of fluorescently labelled Omicron MiniVs (1% Liss Rhod PE) (right). B) Schematic illustration of ACE2 blocking of Omicron MiniVs (left) and representative confocal image of fluorescently labelled Omicron MiniVs (1% Liss Rhod PE) (right). Scale bars are 20 μm.

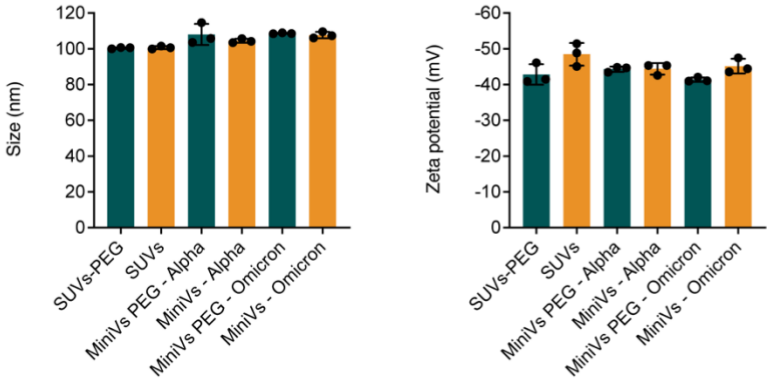

Figure S7. Size and zeta potential measurements of pegylated (green) and non-pegylated (orange) SUVs and MiniVs. Size distribution values were obtained by nanoparticle tracking analysis and zeta potential values were obtained by dynamic light scattering in MilliQ water. Results correspond to the mean ± SD from n = 3 biological replicates in each experimental condition.

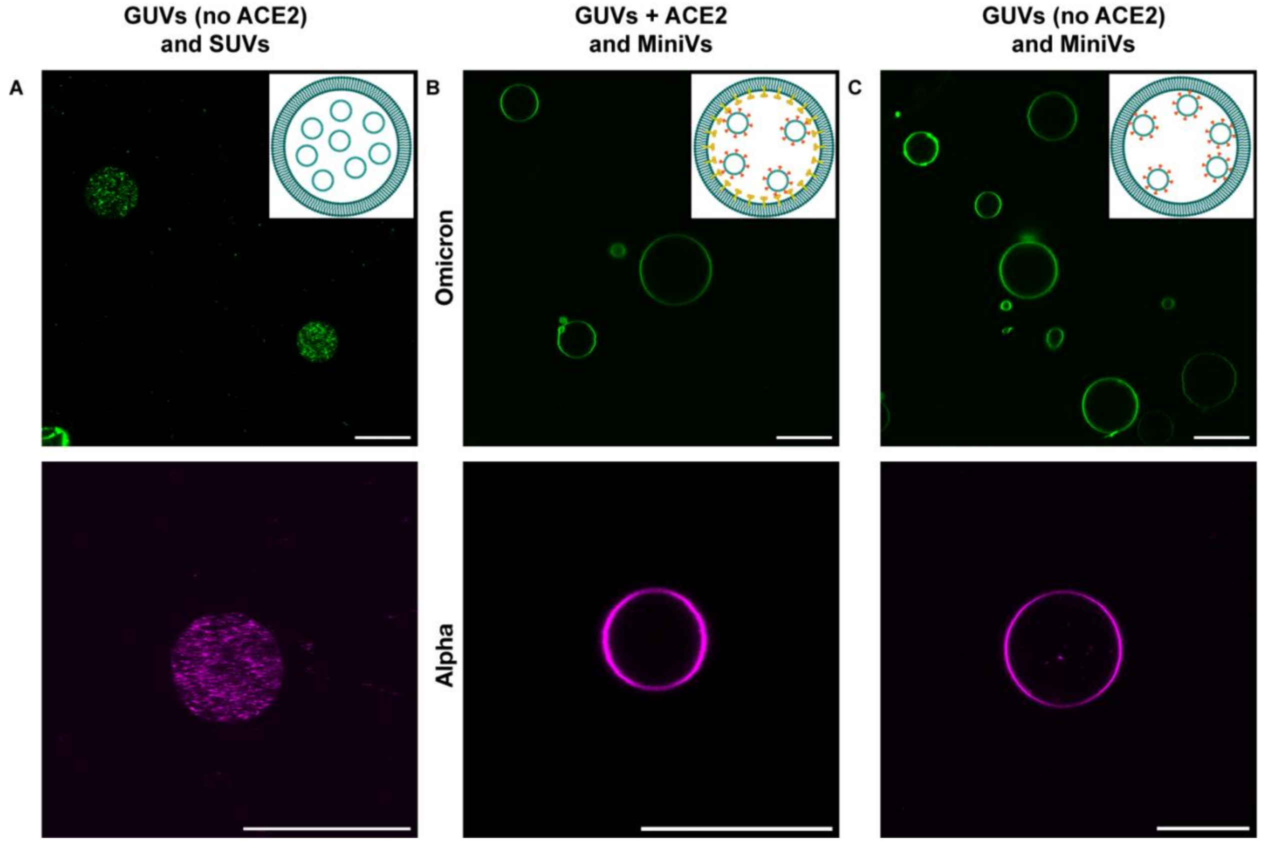

Figure S8. Representative confocal microscopy images of MiniV- or SUV-loaded GUVs. A) Encapsulation of fluorescently labelled SUVs (1% Liss Rhod PE) in non-functionalized GUVs. B and C) Encapsulation of Omicron or Alpha MiniVs (1% Liss Rhod PE) in ACE2-functionalized GUVs (B) or non-functionalized GUVs (C). Scale bars are 20 μm.

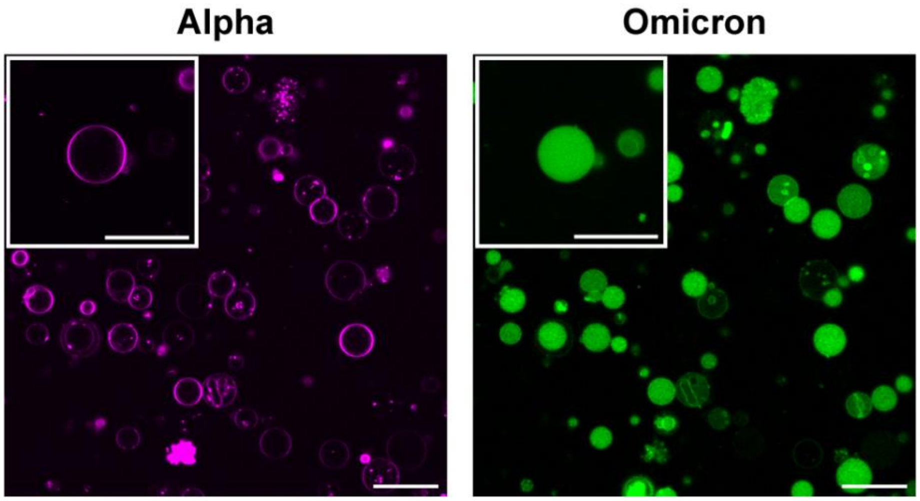

Figure S9. Competition assay to determine Omicron and Alpha MiniVs affinity to the positively-charged non-functionalized GUVs. The fluorescence signals originate from Omicron MiniVs (1% ATTO488 DOPE) (right) or Alpha MiniVs (1% ATTO647 DOPE) (left). The scale bars are 20 μm.

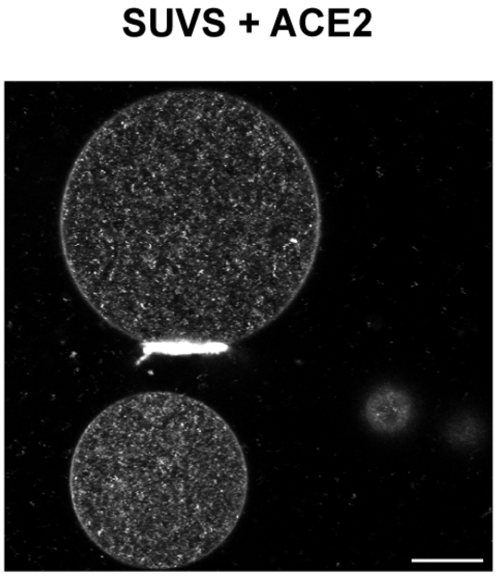

Figure S10. ACE2-functionalized SUVs encapsulated within non-functionalized GUVs. Scale bar is 20 μm.

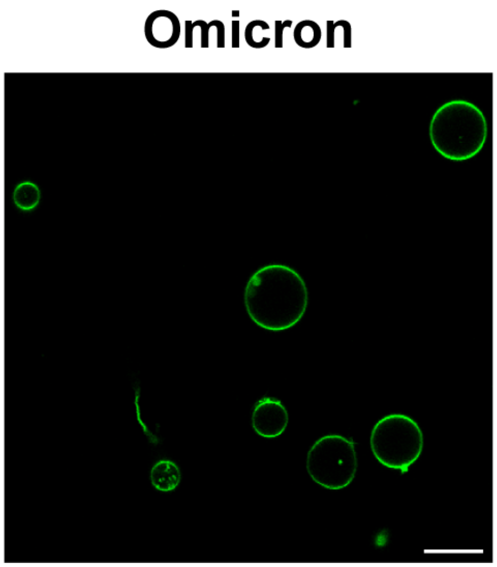

Figure S11. Omicron MiniVs encapsulated in GUVs with a lipid composition lacking NTA-functionalized lipids. Scale bar is 20 μm.

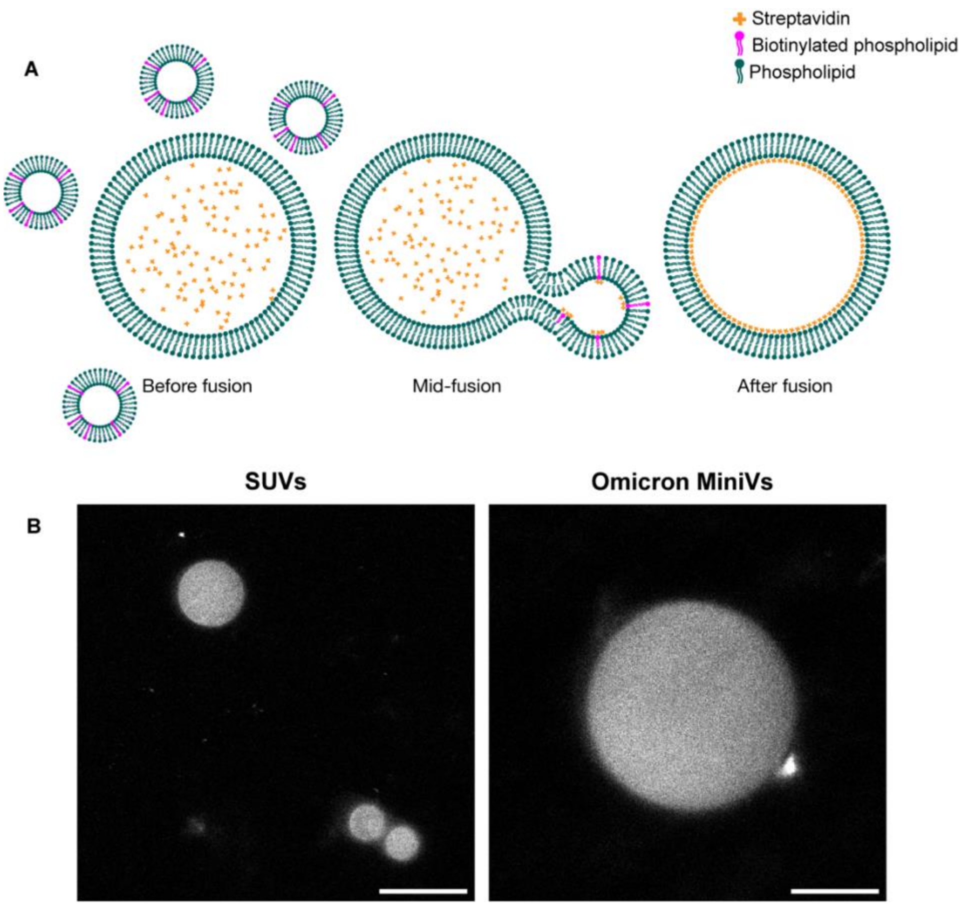

Figure S12. A) Schematic illustration of the fusion experiment. B) Non-fluorescent SUVs (left) or Omicron MiniVs (right) incubated with fluorescent (Alexa-Fluor405) streptavidin-containing GUVs. Scale bars are 20 μm.

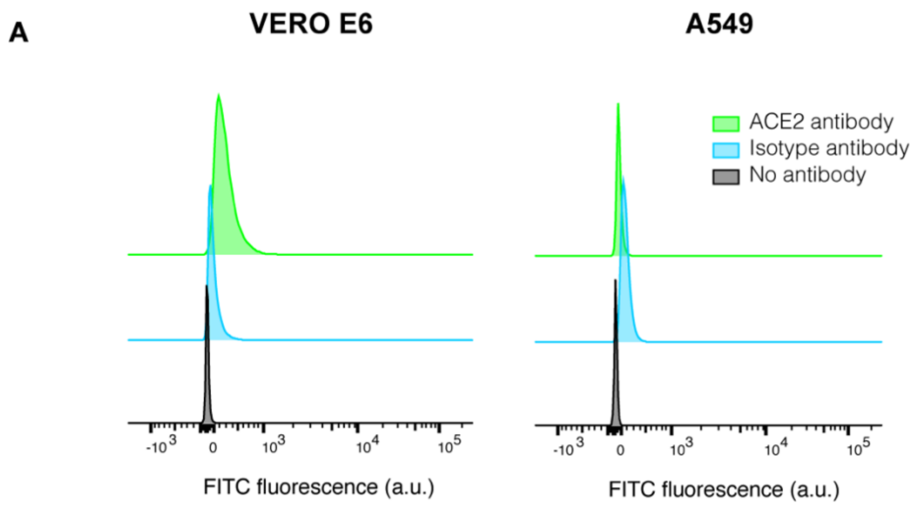

Figure S13. ACE2 receptor staining of VERO E6 and A549 cells.

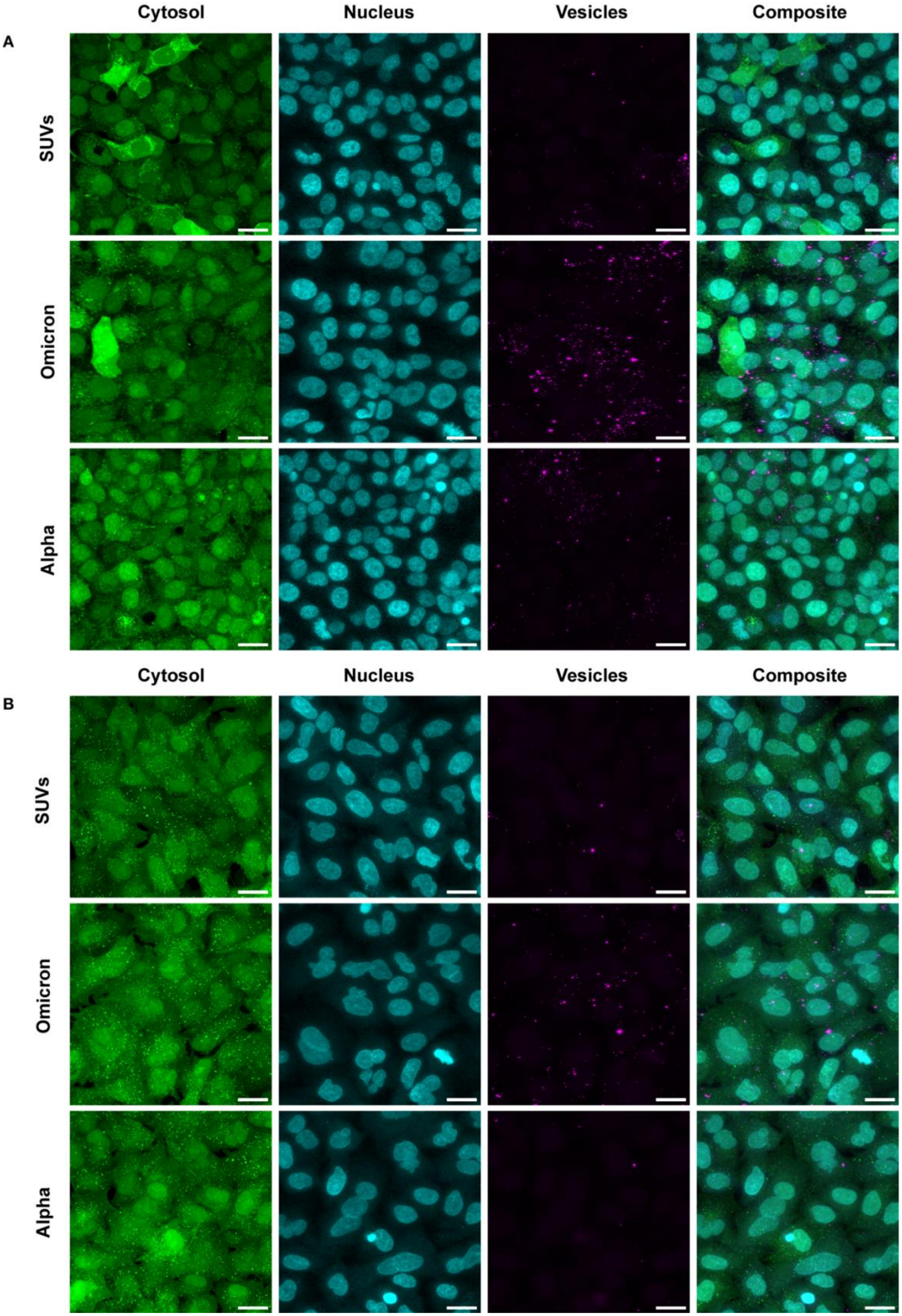
A
B
Cytosol
Nucleus
Vesicles
Composite
SUVs
Omicron
Alpha

Figure S14. A) Representative images of maximal confocal microscopy z-projections of VERO E6 cells incubated with SUVs, Omicron and Alpha MiniVs for 1h. B) Representative images of maximal confocal microscopy z-projections of Vero E6 and A549 cells incubated with Omicron and Alpha MiniVs (magenta) for 1h. Cells were stained with CellTracker™Green CMFDA (cytoplasm, green) and Hoechst 33342 (nucleus, cyan). The scale bars are 20 µm.
